# Consumer purchase intention towards a quick response (QR) code for antibiotic information: an exploratory study

**DOI:** 10.1038/s41538-022-00136-4

**Published:** 2022-04-20

**Authors:** Hollie Bradford, Claire McKernan, Chris Elliott, Moira Dean

**Affiliations:** Institute for Global Food Security, School of Biological Sciences, Queen’s University Belfast, 19 Chlorine Gardens, Belfast, BT9 5DL UK

**Keywords:** Human behaviour, Science, technology and society

## Abstract

Increasing awareness of antimicrobial resistance (AMR) has raised concerns surrounding antimicrobial use (AMU) in food-producing animals and has focused attention towards livestock production free from antibiotic use. As antibiotic-free livestock production proliferates in the UK, there is an increasing need to implement a system, such as the use of a QR code, to provide consumers with reliable antibiotic information while ensuring that animal welfare standards are upheld. Subsequently, this study aims to explore UK consumers’ perceptions and purchase intention towards QR code labelled pork, and to identify determinants of its purchase, incorporating various theoretical constructs from the Theory of Planned Behaviour. Based on results, consumers’ perceptions, perceived control, and attitudes towards QR code labelled pork are the main determinants of purchase intention. QR code labelled pork may offer a suitable alternative to antibiotic-free labelling as it provides consumers with antibiotic information without inadvertently communicating that conventionally produced pork is unsafe.

## Introduction

Over the years, consumer concern over food quality and safety has risen due to a number of food scandals and accidents^1^. Crises such as the Bovine Spongiform Encephalopathy (BSE) in cows, avian influenza, the melamine milk incident in China, and the horse meat in beef scandal, have negatively impacted consumers’ confidence in the food chain and increased worldwide concern over food safety^2–4^. In response to such crises, a food traceability system was established to provide consumers with unambiguous information about the origin and authenticity of the product^5^. Traceability systems have been broadly utilised in the food industries^6–8^ to reduce food safety concerns and to improve marketplace reputation. Food producers have made great efforts to track the flow of food^1^ with many showing motivation to go beyond mandatory traceability requirements^4^, for example, the British pork industry has been at the forefront of developing a DNA/RFID traceability solution incorporating blockchain technology, delivering both knowledge and authenticity assurances to consumers^9^. Despite these advances, various aspects of food production remain under public scrutiny.

Most recently, antimicrobial use (AMU) in food producing animals is one of the most criticised issues in modern pig production^10^. Despite the 2006 EU ban on antibiotics for growth promotion, the dispute surrounding the extent of AMU in livestock production remains unsettled. Such issues are often exacerbated by the media, as a 2015 article from the Guardian, a British national newspaper, reported the discovery of MRSA in pork products sold in supermarkets^11^. Not only was this article about food contamination, but notably it highlighted antibiotic use in pig farming as the root cause of MRSA, and the implications for human health^11^. Subsequently, recent reports of ‘pig-MRSA’ in the media suggest that familiar concerns encompassing biosecurity in agriculture (i.e., food contamination) are converging with those around AMU^11^.

Although the most recent UK One Health Report has revealed that the majority of antibiotics consumed in 2017 were prescribed for human use (491 tonnes; 64%), compared to only 204 tonnes (26%) for use in food-producing animals^12^, AMU in livestock production is still perceived as one of the primary causes for the increase in antimicrobial resistance (AMR)^13^, with many concerns surrounding the transfer of resistant bacteria from animals to humans via the food chain. As a result, there have been proposals to partially or even completely eliminate antibiotic use in agriculture^14^. Completely eliminating antibiotic usage is not only detrimental to animal welfare but the use of ‘antibiotic-free’ or ‘raised without antibiotics’ food labels could give consumers the impression that, by default, conventionally produced foods are unsafe.

While antibiotic use in UK pork production has decreased considerably from 278 mg/population correction unit (PCU) in 2015 to 105 mg/PCU in 2020^15^, quantified antibiotic usage at farm level and specific data surrounding the quantity and patterns of use are not available^16,17^. Additionally, collecting such information presents a variety of challenges including variations in study objectives as investigators may only measure therapeutic use, only non-therapeutic use, or a combination of both^17^. Furthermore, it is unrealistic for farmers to record antibiotic usage for each individual animal, especially those operating at a large scale. To the best of the authors’ knowledge, there is currently no operational system to collect and disseminate antibiotic usage information to consumers at the point of purchase, presenting a unique opportunity for the development of a new system similar to that of a traceability system.

Quick Response (QR) codes are one of the most popular traceability systems having been introduced into the food industry as a two-dimensional barcode^5,18,19^. Scanning the QR code on a smartphone enables easy access to information specific to that product^19^, a concept employed globally and accepted by consumers^18^. QR code usage is estimated to reach 10.1 million in Europe by 2020^20^ and the food traceability market is expected to reach $18,528 million by 2023 with a compound annual growth rate of 9.1%^21^. As such, sector wide research into the application of QR codes is vast, with more research emerging relating to food traceability^4,5,19,22,23^. Research has highlighted the success gained by food traceability systems, as consumers generally link traceability with safety and quality attributes^24,25^, instilling trust in both a certain food product and also in the food system as a whole^1,26^. Specifically, an estimated 92% of consumers revealed a desire to access transparent information on product’s labelling, validating the need for QR codes on food packaging^21^. As a result, several well-known brands such as Nestle, have recently added QR codes onto their best-selling products such as the instant Maggi noodles^27^.

Substantial research surrounding consumer food choice, typically applies a conceptual framework, such as the Theory of Planned Behaviour (TPB)^28^ to explain the antecedents of behaviour^4,29–32^. The TPB has been successfully applied in health and food choice research as intention to perform a behaviour typically precedes, and thereby predicts, the actual behaviour^33^. The TPB postulates that behavioural intention is determined by an individual’s attitude, subjective norm, and perceived behavioural control (PBC)^28^. In addition to theoretical research, various studies have focused on consumers’ willingness-to-pay (WTP) for products that have been produced with increased concern for animal welfare^10,34–37^, particularly as research conducted into consumer preference illustrates that concerns for the wellbeing of livestock is of great interest to them^10,33,38,39^. Indeed, Kehlbacher et al.^35^ found that each month, consumers were willing to increase meat expenditure by 26 and 32% for meat produced with ‘enhanced’ and ‘excellent’ welfare standards, respectively.

In addition, research specific to pig welfare has commonly found that consumers have a positive attitude towards increased welfare standards for pigs^40–42^. However, since the introduction of ‘antibiotic-free’ and associated labels, contradictory findings have emerged. According to Karavolias et al.^43^, when purchasing ‘antibiotic-free’ labelled poultry, consumers believe this label perpetuates high levels of animal welfare. Of consumers who purchase meat products raised without antibiotics, 70% believe that animal health is significantly improved^44^ and that they are promoting good livestock production practices^43^. This is consistent with findings by Goddard et al.^13^ suggesting that consumers are unaware of the negative ramifications that a ban on antibiotic use in livestock production may have on animal welfare. These findings suggest that such labels may be misleading consumers, resulting in further confusion. In addition, it highlights an information gap among consumers in relation to understanding their food labels.

As the use of ‘antibiotic-free’ labelling proliferates, there is an increasing need to implement a system similar to that of a food traceability system to enable consumers access to reliable antibiotic information, providing both quality and safety assurances about the food product, but also ensuring that animal welfare standards are upheld. While providing consumers with the choice to purchase QR code pork, this does not guarantee that all consumers will choose to do so. Food choice is often contingent upon values, food attributes they consider important, and their motivation to use available information^43^.

Therefore, it is necessary to explore consumers’ perceptions and purchase intentions towards QR code pork to characterise consumers response to information as an integral aspect in the development of food marketing and communication strategies^45^. Pork was selected as the test food as the UK pig industry is recognised to be the highest user of antibiotics, believed to use more pro rata than any other livestock production sector^46^. In addition, safety concerns and negative media representation have led to feelings of uncertainty and mistrust among consumers surrounding pork safety^45^.

The objectives of the research were (1) to determine consumers’ purchase intention of QR code labelled pork and the exploratory factors that influence purchase, (2) to identify if the TPB constructs’ ‘attitude’, ‘PBC’, and ‘trust’, influence purchase intention of QR code labelled pork, and (3) to understand consumers’ perceptions of QR code labelled pork. The study will offer insights into how QR code labelled pork influences consumer perceptions and attitude to pork, and how the label influences consumer purchasing decisions.

## Results

### Pork consumption and purchasing behaviour

In total, 1000 participants aged 18–92 years old (M = 46.8, SD = 16.8) completed the survey (see Table 1 for socio-demographic details). The majority of participants were female (51%), educated to university level (27%), and were in full time employment (49%). All respondents shared at least some shopping responsibility. Two out of three participants stated that they purchase pork occasionally (1–4 times a month) and 54% reported occasional pork consumption (1–4 times a month). When purchasing pork, participants considered extrinsic qualities (M = 5.56, SD = 0.97) (i.e., price, quality, quantity, and appearance) as the most important attributes influencing their purchase intention, followed by qualities concerning animal welfare (M = 5.00, SD = 1.13). Marketing qualities (M = 4.77, SD = 1.25) relating to the place of purchase and the brand were the least important attributes. In relation to QR code use, of those respondents with a smartphone, approximately half (51%) have used it to scan a QR code.

**Table 1:** Socio-demographic details and characteristics of the total study sample according to question completion related to QR code one or QR code two.

		Total	QR code 1	QR code 2
		*n* = 1000	*n* = 495	*n* = 505
		%	%	%
**Gender**	Male	49	51	47
	Female	51	49	53
**Age**	18–24 years	12	11	13
	25–34 years	17	17	17
	35–44 years	18	17	19
	45–54 years	18	17	18
	55–64 years	15	16	14
	65+ years	20	22	19
**Social class**	ABC1^1^	51	50	52
	C2DE^2^	49	50	48
**Highest education level**	Primary education	1	1	1
	Secondary education (GCSE or equivalent)	21	21	21
	Secondary education (A-levels or equivalent)	16	17	16
	Vocational or technical qualifications (e.g., HND)	22	22	22
	University level	27	27	27
	Postgraduate level	11	10	11
	Doctorate, post-doctorate or equivalent	2	2	2
**Occupation**	Employed full-time (>30 h per week)	49	49	50
	Employed part-time ≤29 h per week)	14	13	15
	Full-time homemaker	5	6	5
	Unemployed	6	6	6
	Student	5	3	5
	Retired	21	23	19
**Marital status**	Married	51	52	50
	Single (never married)	**27**	28	26
	Widowed	**3**	2	4
	Divorced	5	5	5
	Separated	1	1	1
	Living with partner	13	12	14
**Household income**	Under £6,999 per annum (less than £135 per week)	2	2	3
	£7,000 - £14,999 per annum (£135 - £290 per week)	10	8	12
	£15,000 – £29,999 per annum (£290 - £580 per week)	27	30	24
	£30,000 – £59,999 per annum (£580 - £1,150 per week)	36	34	37
	£60,000 + per annum (£1,150 per week)	17	18	16
	Not sure	2	2	2
	Prefer not to say	6	6	6
**Household size**	1	18	18	19
	2	58	57	59
	3	13	15	11
	4	8	8	8
	5+	3	2	3
	0	72	73	70
**Number of children under 16 in household**	1	15	14	15
	2	10	10	11
	3+	3	3	4
**Frequency of pork purchase**	Daily	1	1	2
	Several times a week	14	12	15
	Several times a month	64	66	62
	Every few months	21	21	21
**Frequency of pork consumption**	Daily	1	1	2
	Several times a week	31	32	30
	Several times a month	54	53	55
	Every few months	14	14	13

^1^High social class: includes professional, managerial, technical, and skilled non-manual occupations.

^2^Low social class: includes skilled manual, partly skilled, and unskilled occupations.

### Consumer perceptions, intention to buy and willingness to pay towards QR code labelled pork

**Fig. 1:**
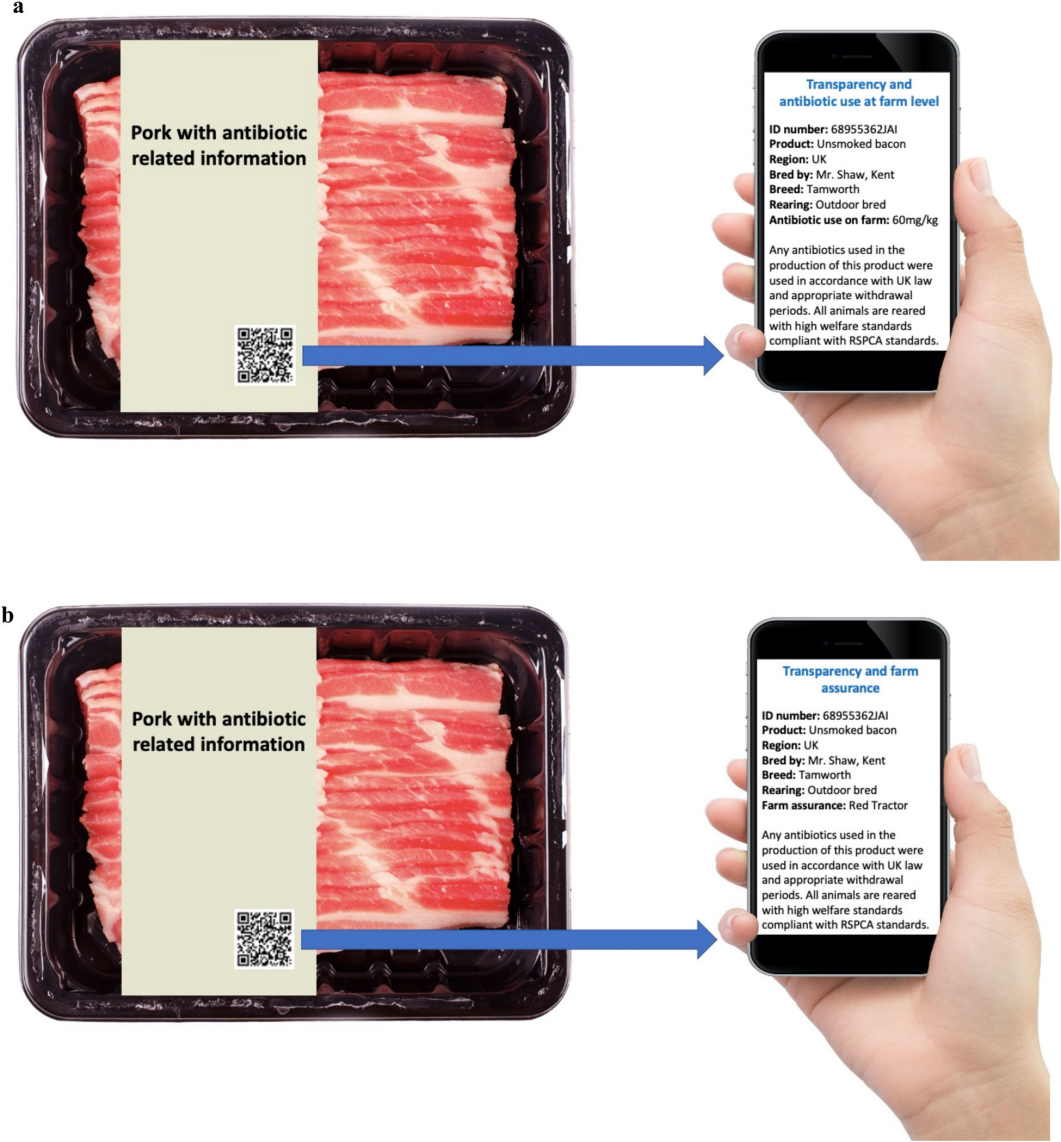
Respondents were shown a visual aid, specific to QR code one (antibiotic usage labelled pork) or QR code two (farm assurance labelled pork), depicting the type of information which could be retrieved upon scanning the package QR code. **a** QR code one (antibiotic usage label). **b** QR code two (farm assurance label). Figure created with Shutterstock and Pixabay.

After seeing a visual aid of QR code labelled pork (Fig. 1) participants reported a slightly favourable attitude (QR1: 4.79 and QR2: 4.77) with a high trust (QR1: 4.90 and QR2: 4.91) towards the QR code product (Table 2). Participants claimed purchasing QR code labelled pork, in comparison to traditional pork, to be wise, beneficial, and made them feel “good” and “pleased.” Behavioural beliefs towards the QR code product varied as participants recorded a strong belief that QR code pork would be more expensive than traditional pork (item scored above 5 in both sub-groups); however, a negative score was recorded in relation to ease of locating the product, indicating that participants believe that QR code labelled pork will not be easy to find in supermarkets (QR1: 3.87 and QR2: 3.84). With regard to trust of the product, participants specifically thought that QR code labelled pork will provide an assurance that antibiotics have been used on the animal responsibly during production (QR1: 4.93 and QR2: 4.98).

**Table 2:** Standardised factor loadings, Cronbach’s alpha, and Mean (SD) response of questionnaire variables which were scored on a 7-point Likert-type scale (1 = ‘strongly disagree”, 7 = “strongly agree”) for each sub-group (QR code one and QR code two).

Variables *Items*	Alpha	Factor loadings	Mean (SD) response	
			QR code 1	QR code 2
			*n* = 495	*n* = 505
**Attitude**	0.93		4.79 (1.06)	4.77 (0.99)
*Buying QR code labelled pork instead of traditional pork now available in supermarkets would make me feel:*				
Scale: very bad – very good		0.91	4.75 (1.12)	4.78 (1.04)
Scale: very displeased – very pleased		0.87	4.72 (1.17)	4.68 (1.08)
I think that buying QR code labelled pork instead of traditional pork is:				
Scale: very foolish – very wise		0.86	4.86 (1.21)	4.83 (1.19)
Scale: very harmful – very beneficial		0.85	4.82 (1.19)	4.77 (1.09)
**PBC**	0.93		4.76 (1.21)	4.82 (1.18)
*Regarding the additional information about antibiotic use of QR code labelled pork (obtained via the code):*				
it will be easy to find the antibiotic information		0.81	4.72 (1.50)	4.76 (1.47)
I am confident that I’ll find the antibiotic information		0.84	4.70 (1.49)	4.72 (1.48)
I will be able to find the antibiotic information without help from others		0.84	4.80 (1.61)	4.84 (1.56)
it will be easy to understand the antibiotic information (the type of drug and amount in ml)		0.92	4.62 (1.47)	4.74 (1.45)
I am confident that I’ll understand the antibiotic information (the type of drug and amount in ml)		0.90	4.64 (1.52)	4.75 (1.51)
I will be able to understand the antibiotic information without help from others		0.89	4.67 (1.52)	4.77 (1.50)
I would prefer to see a rating system or colour coding to indicate if antibiotic use is high rather than a figure in ml (e.g., similar to the traffic light rating system)		0.34	5.26 (1.41)	5.18 (1.33)
even if I don’t understand the information, I can use this label as a form of assurance that antibiotics have been used responsibly (withdrawn & safe)		0.64	4.67 (1.42)	4.81 (1.31)
**Trust**	0.94		4.90 (1.22)	4.91 (1.28)
*I trust:*				
that QR code labelled pork can provide accurate and reliable information surrounding antibiotic use during production		0.92	4.92 (1.31)	4.89 (1.38)
that the information about adherence to the withdrawal period is reliable on QR code labelled pork		0.91	4.85 (1.27)	4.87 (1.33)
that QR code labelled pork will provide an assurance that antibiotics have been used on the animal responsibly		0.90	4.93 (1.33)	4.98 (1.35)
**Perceptions of QR code**	0.92		4.42 (1.31)	4.55 (1.31)
*Based on the idea of QR code labelled pork becoming available:*				
I believe this QR code would be useful		0.84	4.57 (1.73)	4.76 (1.73)
I would like to see this QR code on pork products		0.86	4.52 (1.72)	4.64 (1.73)
Seeing this QR code on foods will assure me that antibiotics have been used on the animal responsibly		0.85	4.46 (1.63)	4.60 (1.64)
I would eat meat from animals which had antibiotics knowing that the animal hasn’t suffered		0.56	4.63 (1.44)	4.72 (1.43)
Buying products with this QR code will reduce my risk of consuming antibiotics		0.81	4.35 (1.55)	4.46 (1.50)
Buying products with this QR code will reduce my chances of getting AMR		0.80	4.24 (1.54)	4.32 (1.53)
Buying products with this QR code will help me not worry as much about AMR		0.81	4.18 (1.56)	4.32 (1.50)
**Beliefs (quality)**	0.88		4.19 (1.16)	4.21 (1.20)
QR code labelled pork will likely be tastier		0.83	4.13 (1.34)	4.17 (1.38)
QR code labelled pork will likely be easier to find		0.61	3.87 (1.37)	3.84 (1.33)
QR code labelled pork will likely be of more satisfying quality		0.88	4.26 (1.33)	4.29 (1.42)
QR code labelled pork will likely be safer to eat		0.88	4.50 (1.40)	4.48 (1.41)
**Beliefs (animal welfare)**	0.83		4.51 (1.32)	4.55 (1.31)
QR code labelled pork will likely be healthier		0.79	4.19 (1.44)	4.26 (1.52)
QR code labelled pork will likely have higher animal welfare standards		0.82	4.66 (1.38)	4.65 (1.36)
QR code labelled pork will likely be free from antibiotics		0.72	4.35 (1.52)	4.44 (1.47)
**Beliefs (expense)**	-		5.00 (1.31)	5.06 (1.31)
QR code labelled pork will likely be more expensive		0.41		
**Intention**	0.95		4.20 (1.46)	4.29 (1.51)
*If pork products with this QR code become available:*				
I intend to buy them		0.91	4.23 (1.48)	4.34 (1.55)
I will look for them		0.93	4.25 (1.59)	4.36 (1.65)
It will be important for me to buy them		0.92	3.98 (1.56)	4.13 (1.62)
I will buy them to find out more about animal welfare standards		0.88	4.34 (1.61)	4.32 (1.65)
**Generalised trust**	0.93		4.57 (1.15)	4.59 (1.19)
*Most people:*				
are basically honest		0.92	4.51 (1.32)	4.51 (1.38)
are trustworthy		0.94	4.51 (1.27)	4.51 (1.35)
are basically good and kind		0.86	4.70 (1.22)	4.77 (1.24)
are trustful of others		0.78	4.58 (1.22)	4.56 (1.26)
**Purchasing habits (extrinsic qualities)**	0.75		5.60 (0.93)	5.52 (1.01)
*Please rate the following based on their level of importance when purchasing pork:*				
Price		0.52	4.97 (1.45)	4.86 (1.49)
Quality (for example, taste/flavour/freshness)		0.73	6.08 (1.27)	5.95 (1.41)
Quantity (for example, size)		0.67	5.50 (1.09)	5.49 (1.19)
Appearance (for example, colour/texture)		0.71	5.81 (1.14)	5.77 (1.17)
**Purchasing habits (animal welfare qualities)**	0.88		5.00 (1.17)	5.02 (1.09)
Origin (for example, local, British, EU)		0.51	5.28 (1.46)	5.23 (1.45)
Antibiotics used		0.66	5.03 (1.60)	5.07 (1.52)
Organic (or other assurance certificate)		0.69	4.41 (1.68)	4.49 (1.65)
Animal welfare practices		0.90	5.32 (1.46)	5.33 (1.40)
Healthiness/nutritional content		0.49	5.26 (1.36)	5.33 (1.25)
Environmental friendliness		0.87	5.04 (1.43)	5.06 (1.41)
The type of packaging		0.54	4.52 (1.52)	4.61 (1.48)
**Purchasing habits (marketing qualities)**	0.58*		4.80 (1.24)	4.75 (1.25)
Place of purchase		0.51	5.08 (1.30)	4.96 (1.36)
The brand		0.91	4.51 (1.49)	4.53 (1.46)
**Perception of AMU (personal concern)**	0.65		5.04 (1.19)	5.05 (1.20)
*When considering antibiotic use:*				
I am concerned that AMR will affect me one day		0.71	4.57 (1.71)	4.45 (1.74)
too many antibiotics from the doctor can cause AMR		0.67	5.53 (1.48)	5.59 (1.39)
if I have AMR, I will not be able to treat illness		0.50	5.02 (1.49)	5.12 (1.49)
**Perception of AMU (animal welfare standards)**	0.75*		5.71 (1.25)	5.67 (1.21)
it is important to me that animal welfare standards are adhered to when purchasing meat		0.85	5.65 (1.35)	5.59 (1.30)
it is important to me that the pork I buy has been produced in a way that the animal has experienced as little pain as possible		0.88	5.76 (1.32)	5.75 (1.30)
**Perception of AMU (animal usage acceptance)**	0.71		4.20 (1.19)	4.30 (1.06)
I would be willing to consume meat from animals treated with antibiotics		0.68	4.31 (1.43)	4.30 (1.38)
overall, the use of animal antibiotics delivers more benefits than harm		0.70	4.09 (1.42)	4.25 (1.24)
the use of antibiotics in livestock cannot be seriously harmful, otherwise usage would be banned		0.64	4.19 (1.55)	4.36 (1.42)
**Perception of AMU (animal concern)**	0.55		4.08 (1.11)	4.17 (1.07)
using antibiotics in livestock makes them less effective in humans		0.50	4.61 (1.52)	4.59 (1.41)
antibiotics should never be used in livestock production, even in medical need, since it is critical to maintain useful antibiotics for public health use		0.61	4.26 (1.52)	4.37 (1.45)
I consider domestic pets to be a potential source of transfer of AMR		0.55	3.35 (1.54)	3.55 (1.57)

*Inter-item correlation (*p* < 0.01).

Items removed from the measure on the basis of exploratory factor analysis were as follows: ‘using antibiotics in livestock makes them less effective in humans’, ‘antibiotics should never be used in livestock production, even in medical need, since it is critical to maintain useful antibiotics for public health use’, and ‘I consider domestic pets to be a potential source of transfer of AMR’.

In both QR code sub-groups, participants showed a moderately high level of PBC (QR1: 4.76 and QR2: 4.82) stating they were able to find and understand the antibiotic-related information embedded in the QR code. However, they strongly indicated (QR1: 5.26 and QR2: 5.18) that they preferred a rating system or colour coding to indicate if antibiotic use is high rather than a figure in ml (i.e., similar to the traffic light rating system providing nutritional information on food). Participants perceived personal risk and consideration towards animal welfare standards to be high (all items scored above 5 in both sub-groups). Participants thought that too many antibiotics from the doctor can cause AMR and that AMR will interfere with disease treatment. In addition, adherence to animal welfare standards and ensuring animals do not experience pain were considered important. Contrastingly, participants were neutral in their acceptance of AMU in livestock production (QR1: 4.20 and QR2: 4.30) and held negative perceptions towards domestic pets acting as a potential source of AMR transfer (QR1: 3.35 and QR2: 3.55, *p* = 0.043); indicating that they do not consider domestic animals to act as a reservoir or spread resistant bacteria.

Participants held moderately positive perceptions (QR1: 4.42 and QR2: 4.55) towards QR code labelled pork and showed a general high level of knowledge towards EU regulations (M = 3.29 out of a possible 5, SD = 1.01). However, awareness of AMR was limited; while the majority had heard of AMR (52%), only 38% of respondents knew what AMR is.

Participants in both sub-groups were neutral in their intention to buy QR code labelled pork (QR1: 4.20 and QR2: 4.29); with no significant differences between antibiotic usage and farm assurance labelled pork. Conversely, when examining the willingness to purchase antibiotic usage labelled pork; 34.8% of the sample were not willing to pay more, and of the 65.2% who were willing to pay extra, on average they were willing to pay ~10% more. For farm assurance labelled pork, 35.2% of the sample were unwilling to pay a price premium and of the 64.8% of those who would pay; on average they were willing to pay ~15% more.

### Predicting intention to buy QR code labelled pork

All exploratory constructs except knowledge of EU regulations and awareness of AMR, correlated significantly with intention to purchase QR code labelled pork in both sub-groups (see Table 3). Perception of QR code had the strongest relationship with intention, indicating that those with more positive perceptions towards QR code labelled pork were more likely to intend to purchase it. Moderately positive correlations were also observed within both sub-groups between intentions and the following constructs: attitude, PBC, trust, and perceptions toward animal welfare standards.

**Table 3:** Correlations between intention and other constructs contained within the QR code one and QR code two models.

QR code 1	1	2	3	4	5	6	7	8	9	10	11	12	13	14	15
**(Antibiotic usage label)**															
1. Intention	—														
2. Attitude	0.70**	—													
3. PBC	0.60**	0.58**	—												
4. Trust	0.58**	0.61**	0.63**	—											
5. Perception of QR code	0.83**	0.69**	0.64**	0.66**	—										
6. Generalised trust	0.25**	0.25**	0.27**	0.35**	0.30**	—									
7. Perception of AMU (personal concern)	0.15**	0.24**	0.25**	0.20**	0.15**	0.24**	—								
8. Perception of AMU (animal usage acceptance)	0.13**	0.24**	0.19**	0.27**	0.23**	0.16**	−0.04	—							
9. Perception of AMU (animal welfare standards)	0.32**	0.30**	0.37**	0.34**	0.29**	0.32**	0.45**	−0.03	—						
10. Knowledge of EU regulations	0.01	0.04	0.03	0.14**	0.05	0.17**	0.03	0.05	0.04	—					
11. Awareness of AMR	−0.05	0.06	0.06	0.01	−0.02	0.03	0.26**	−0.07	0.11*	0.03	—				
12. Age	−0.10*	−0.08	−0.16**	−0.004	−0.10*	0.13**	0.03	−0.15**	0.07	0.16**	−0.06	—			
13. Education	0.03	0.08	0.03	−0.05	0.01	0.04	0.06	0.05	−0.002	0.03	0.19**	−0.18**	—		
14. Gender	−0.02	0.02	0.04	0.03	0.02	−0.06	0.08	−0.02	0.05	−0.06	0.03	−0.18**	−0.03	—	
15. Socioeconomic status	−0.10*	−0.12**	−0.15**	−0.09*	−0.13**	−0.06	−0.08	−0.15**	0.000	0.05	−0.12**	0.19**	−0.17**	0.06	—
**QR code 2 (Farm assurance label)**															
1. Intention	—														
2. Attitude	0.72**	—													
3. PBC	0.62**	0.55**	—												
4. Trust	0.64**	0.63**	0.62**	—											
5. Perception of QR code	0.85**	0.69**	0.61**	0.70**	—										
6. Generalised trust	0.22**	0.19**	0.23**	0.26**	0.26**	—									
7. Perception of AMU (personal concern)	0.28**	0.23**	0.24**	0.22**	0.24**	0.08	—								
8. Perception of AMU (animal usage acceptance)	0.14**	0.14**	0.18**	0.12**	0.18**	0.20**	0.06	—							
9. Perception of AMU (animal welfare standards)	0.35**	0.31**	0.32**	0.35**	0.34**	0.19**	0.35**	0.05	—						
10. Knowledge of EU regulations	−0.06	−0.02	−0.007	0.01	−0.002	0.02	−0.07	−0.02	0.01	—					
11. Awareness of AMR	0.004	0.04	0.05	0.002	−0.02	−0.02	0.31**	−0.03	0.09*	−0.03	—				
12. Age	−0.13**	−0.10*	−0.15**	−0.08	−0.12**	0.14**	−0.06	−0.14**	0.15**	0.11*	−0.07	—			
13. Education	0.03	0.07	0.02	0.04	−0.004	−0.01	0.09	−0.04	−0.02	0.02	0.14**	−0.10*	—		
14. Gender	0.12**	0.09	0.05	0.10*	0.12**	−0.02	0.12**	−0.03	0.09*	−0.10*	0.07	−0.27**		—	
15. Socioeconomic status	−0.12**	−0.13**	−0.14**	−0.09*	−0.11*	0.03	−0.08	−0.04	0.01	−0.01	−0.15**	0.30**	−0.31**	−0.08	—

*p* < 0.05*; < 0.01**; bold numbers highlight significance.

The regression analysis revealed that consumers’ intention to buy QR code labelled pork is driven by their attitude (both sub-groups), PBC (both sub-groups), perception of QR codes (both sub-groups), personal concern perceptions (farm assurance label sub-group), perceptions towards animal AMU acceptance (antibiotic usage label sub-group), perceptions towards animal welfare standards (antibiotic usage label sub-group), knowledge of EU regulations (farm assurance label sub-group), and awareness of AMR (antibiotic usage label sub-group). Together these exploratory variables account for 73% (based on R^2^_adj_) of the variance in intention to purchase pork labelled with antibiotic use and 77% of the variance in intention to purchase farm assured labelled pork (Table 4).

**Table 4:** Standardised regression coefficients (ß) for both model 1 constructs and model 2 extended socio-demographic constructs from regression analysis predicting consumers’ intention to buy QR code labelled pork.

Variables	Model 1		Model 2	
	QR code 1	QR code 2	QR code 1	QR code 2
Attitude^1^	0.25***	0.22***	0.25***	0.22***
PBC^1^	0.08*	0.11***	0.07*	0.11***
Trust^1^	−0.04	−0.04	−0.03	−0.04
Perception of QR code^1^	0.64***	0.64***	0.64***	0.64***
Generalised trust^1^	−0.003	−0.004	−0.004	0.001
Perception of AMU (personal concern)^1^	−0.03	0.06*	−0.03	0.05*
Perception of AMU (animal usage acceptance)^1^	−0.08**	−0.03	−0.08**	−0.03
Perception of AMU (animal welfare standards)^1^	0.06*	0.02	0.07*	0.02
Knowledge of EU regulations^2^	−0.02	−0.05*	−0.02	−0.04
Awareness of AMR^3^	−0.06**	−0.02	−0.07**	−0.02
Age	—	—	−0.02	−0.02
Education	—	—	0.01	0.006
Gender	—	—	−0.04	0.008
Socioeconomic status	—	—	0.008	−0.004
R^2^_*adj*_	0.73	0.77	0.73	0.77
Model F	136.42***	165.91***	97.72***	117.96***
ΔR^2^	—	—	0.002	0.001
D*f*	484	494	480	490

Model 2 refers to the extended model with socio-demographics.

*p* < 0.05*; < 0.01**; < 0.001***; bold text highlights significance.

^1^ Mean of variable items measured on a 7-point Likert scale: higher scores indicative of stronger (i.e., more positive) levels of the construct.

^2^ Knowledge scale 0–5 based on 5 true/false questions. 0 = low knowledge and 5 = high knowledge.

^3^ Awareness of AMR scale 0–1 based on yes/no questions where 0 = low awareness and 1 = high awareness.

That is, having a more favourable attitude towards the labelled product (ß = 0.25 and ß = 0.22 for the antibiotic usage and farm assurance label sub-group, respectively), a higher PBC for finding and understanding the antibiotic information (ß = 0.08 and ß = 0.11 for the antibiotic use and farm assurance label sub-group, respectively), and having more favourable perceptions towards the QR code (ß = 0.64 and ß = 0.64 for the antibiotic usage and farm assurance label sub-group, respectively), were associated with a greater intention to purchase it. Additionally, having an increased level of personal concern towards AMU (ß = 0.06) was associated with a greater intention to buy farm assurance labelled pork; while, having favourable perceptions towards animal welfare standards (ß = 0.06) was associated with an increased intention to buy antibiotic usage labelled pork. While perceptions towards animal AMU acceptance (ß = −0.08 for the antibiotic usage label sub-group), awareness of AMR (ß = −0.06 for the antibiotic use label sub-group), and knowledge of EU regulations (ß = −0.05 for the farm assurance label sub-group) were also determinants of purchase intention, they had a negative influence on intention to purchase QR code labelled pork. Therefore, having less favourable perceptions towards the acceptance of AMU in animals, lacking awareness towards AMR, and lacking knowledge towards EU regulations, were associated with a greater intention to purchase QR code pork.

**Fig. 2:**
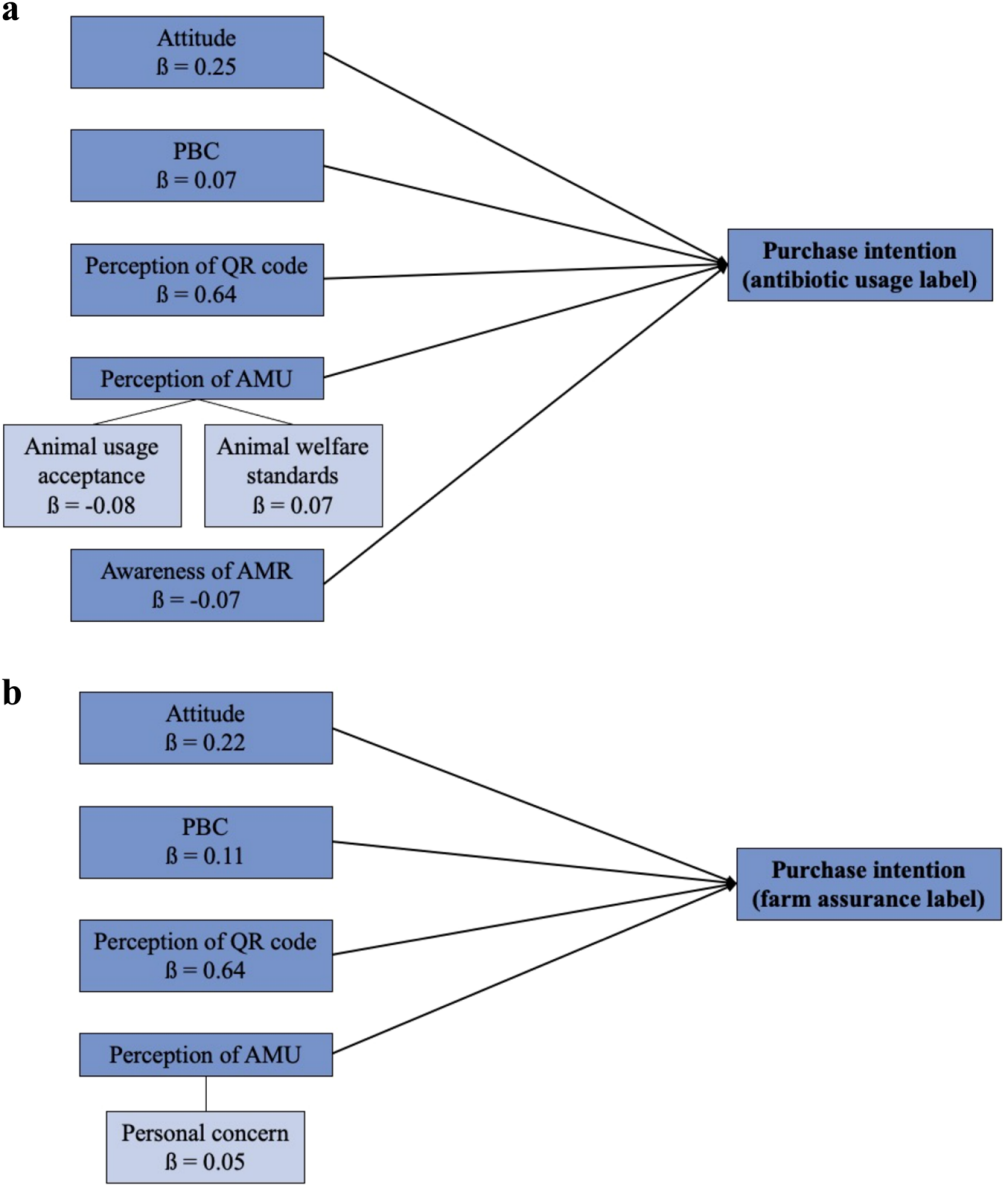
Final regression model showing the exploratory factors influencing intention to purchase QR code labelled pork. **a** QR code one (antibiotic usage label). **b** QR code two (farm assurance label).

When the model was extended with socio-demographic factors in the hierarchical multiple regression, the explained variance (based on R^2^_adj_) in intention to purchase labelled pork remained constant at 73% for the antibiotic usage label sub-group (*p* < 0.001) and 77% for the farm assurance label sub-group (*p* < 0.001). That is, when the model was extended with age, gender, education, and socioeconomic status (SES), socio-demographic characteristics explained little variance in the prediction of purchase intention. Attitude (ß = 0.25), PBC (ß = 0.07), perception of QR code (ß = 0.64), acceptance of animal AMU (ß = −0.08), perceptions towards animal welfare standards (ß = 0.07), and awareness of AMR (ß = −0.07) remained significant determinants of intention to purchase antibiotic usage labelled pork (see Fig. 2a). Comparably, attitude (ß = 0.22), PBC (ß = 0.11), perception of QR code (ß = 0.64), and personal concern towards AMU (ß = 0.05) were all still significant drivers of intention to purchase farm assurance labelled pork, however, awareness of AMR was no longer a predictor (see Fig. 2b).

### Explaining consumer attitudes

To gain an understanding of behavioural beliefs influencing attitude towards QR code labelled pork, the behavioural beliefs were regressed with attitude, as postulated by the TPB. Results revealed that behavioural beliefs relating to product quality (both sub-groups) and animal welfare (farm assurance label sub-group) influence consumer attitude towards labelled pork. Together these exploratory variables account for 37% (based on R^2^_adj_) of the variance in attitude towards antibiotic usage labelled pork and 40% of the variance in attitude towards farm assurance labelled pork (Table 5). Quality attributes were the main determinants of attitude in each sub-group (ß = 0.52 and ß = 0.40 for the antibiotic usage and farm assurance label sub-group, respectively); however, behavioural beliefs on the expense of QR code pork was not a predictor of attitude.

**Table 5:** Standardised regression coefficients (ß) for behavioural beliefs from regression analysis predicting consumers’ attitude of QR code labelled pork for each sub-group (QR code one and QR code two).

Beliefs	Standardised coefficients	
	QR code 1	QR code 2
Quality attributes	0.52*	0.40*
Animal welfare	0.09	0.28*
Expense	0.06	−0.002
R^2^_*adj*_	0.37	0.40
Model F	96.69*	111.17*
D*f*	491	501

*p* < 0.001*; bold text highlights significance.

## Discussion

To our knowledge this is the first study to examine the use of a QR code to deliver antibiotic usage information related to food products to consumers. Specifically, this study identified the influence of individual-level variables (i.e., socio-demographic, and psychological) and constructs from the TPB on intention to purchase QR code labelled pork in a representative sample of adults in the UK.

Overall, the exploratory models showed that psychological contributors to intention to buy QR code pork are somewhat different between each sub-group (antibiotic usage and farm assurance labels). Based on the literature it was expected that the presence of a QR code would influence consumer purchase intention as food packaging with less information is more attractive and considered to be a product of higher quality^47,48^. This study shows that the exploratory models account for 73% (based on R^2^_adj_) of the variance in intention to purchase pork labelled with antibiotic usage information and 77% of the variance in intention to purchase farm assured labelled pork.

Respondents’ attitude and PBC towards QR code pork were strong positive determinants of purchase intention across both labelling sub-groups. Generally, respondents reported a slightly favourable attitude towards QR code pork, with younger participants presenting significantly more positive attitudes towards traceable pork. This finding is in line with Veeman and Li^49^ which showed that older participants perceived more risks in food safety than younger participants; possibly explaining why the younger sample population viewed purchasing QR code pork as more beneficial and thus, as something that makes them feel good and pleased. As a construct of the TPB, attitude is often identified as a determinant of purchase intention^32^. Specifically, Spence et al.^4^ found that attitude had the strongest relationship and was the main determinant of intention to buy both traceable beef steak and mince steak, while McCarthy and colleagues^50^ established that consumers’ attitudes towards pork consumption influenced their intention to consume pork. The TPB postulates that behavioural beliefs influence attitude^29^, and the present study shows that, with the exception of the belief that QR code pork will likely be more expensive, beliefs surrounding product quality (both labelling sub-groups) and animal welfare (farm assurance label sub-group) contribute 37% of the variance in attitude towards intention to buy antibiotic usage labelled pork and 40% towards the purchase intention of farm assurance labelled pork. As attitude is one of the main predictors of intention across both labelling sub-groups, behavioural beliefs should be incorporated into marketing campaigns, with a particular focus on product quality.

In correspondence with their attitude, respondents also reported moderately positive PBC, perceiving themselves to have the ability to find and use the QR code and understand the antibiotic-related information presented. Despite this, respondents in both label sub-groups strongly indicated that they would prefer a rating or colour coded system related to antibiotic usage, similar to that of the traffic light system, rather than usage figures and data. It is possible that this implies a lack of consumer understanding of usage data, however, the traffic light system is an effective tool for conveying complex information^51^; thus, this finding can be used for marketing purposes to ensure that information transfer is both desirable and user friendly. Additionally, as PBC is positively associated with intention to buy QR code pork, increasing consumers usability should further increase their PBC and thus, their intention to purchase QR code pork.

While participants conveyed neutral perceptions towards QR code pork, perception was the main determinant of purchase intention in each sub-group. This result corroborates with other studies, as Rahnama and colleagues^3^ identified that consumers’ perceptions had a significant influence on choosing antibiotic-free chicken, and Schleenbecker and Hamm^52^ uncovered that perception has an important role in purchasing organic foods. However, based on the finding that consumers’ perceptions of QR code pork are only moderately positive, it is clear that there is considerable scope for improving UK consumers’ perceptions; thus, the introduction of marketing communications may be a useful strategy to provide consumers with the benefits of QR codes for accessing traceability information and thereby promote its use.

An interesting finding from this study is that respondents’ perceptions towards AMU practices vary and therefore, while they were found to influence purchase intention, certain perceptions had a positive influence while others had a negative association with purchase intention. Moreover, the perceptions that were found to influence purchase intention additionally varied between each label sub-group. Respondents personal risk concerns surrounding AMU and their consideration towards animal welfare were high. Livestock production free from pain and adherence to animal welfare standards when purchasing meat was important to respondents; however, at the same time, they reported a high level of concern towards contracting AMR, particularly in relation to antibiotics prescribed by a doctor. Overall, in the exploratory model for the antibiotic usage label sub-group, having favourable perceptions towards animal welfare standards was associated with an increased purchase intention, whereas, in the farm assurance label sub-group respondents personal concern towards AMU was associated with a greater purchase intention. It is possible that the former finding suggests that consumers who are more considerate of high animal welfare standards have a greater interest in the antibiotic usage label and accessing exact AMU data instead of the farm assurance label, as it was not a predictor within the farm assurance label sub-group. In comparison to respondents more favourable views, perceptions surrounding the acceptance of animal AMU were moderate and this was reflected in the model for the antibiotic usage label sub-group as it was found to have a negative influence on intention. Consequently, possessing less favourable perceptions towards the acceptance of AMU in animals is associated with a greater intention to purchase. This result was surprising as while perceptions towards animal AMU were more neutral, respondents indicated that they were, nonetheless, positively accepting of AMU in livestock. This finding suggests that more research is required to supplement this preliminary result before a definitive conclusion is reached as to the potential role of this construct in the model of purchase intention towards QR code pork.

Additionally, the exploratory models identified respondent’s awareness of AMR as a determinant of intention to purchase antibiotic usage labelled pork, and knowledge of EU regulations as a determinant of intention to buy farm assurance labelled pork; again, uncovering a negative influence in both sub-groups on intention to buy QR code pork. Previous research has also shown that consumers’ awareness is one of the most important factors in choosing healthy food products^53,54^ and antibiotic-free chicken^3^; however, dissimilar to this study, awareness was found to have a positive influence on intention. The present study determined that respondents generally lack awareness of AMR as just over one third of consumers know what AMR is, yet, as previously discussed, respondents perceive high personal risks associated with AMR. Similarly, research conducted by Public Health England^55^ revealed that misunderstandings about antibiotics persists in the minds of a significant proportion (44%) of the general public, with respondents showing uncertainty around concepts such as carriage of resistant bacteria and whether resistance is caused by taking antibiotics. Although consumers do not fully understand the concept of AMR, it can be speculated that once they were exposed to the QR code product in our study, due to unfamiliarity, they may equate it with increased qualities and automatically assume it is a superior product. Therefore, this may explain why a lack of awareness towards AMR is associated with a greater intention to buy QR code pork. While this finding can be used in the development of consumer campaigns to increase AMR awareness, it must be considered that this rationale is speculation and thus, merits further research.

When extended with socio-demographic characteristics, the predictive power of the exploratory model remained constant at 73% for the antibiotic usage label sub-group (*p* < 0.001) and 76% for the farm assurance label sub-group (*p* < 0.001). Various studies have uncovered a link between socio-demographic characteristics and purchase intention, as Pelletier and colleagues^56^ identified that gender has a positive impact on buying organic food, and Zhang et al.^1^ also observed that females had a higher likelihood of purchasing traceable pork and oil, than males. Additionally, previous studies have shown that age has a significant influence on purchasing healthy food products^56^ whereby younger consumers are more likely to purchase traceable food products than other age groups^1^ and are also more likely to pay extra for meat with reduced antibiotics^13^. By contrast in this study the socio-demographics factors such as gender, age, education, and SES had no significant influence on intention to purchase QR code labelled pork.

In the final model, perception was the main determinant of intention to purchase each QR code product, followed by attitude. As a construct of the TPB, attitude has been identified in various studies as a strong precedent of behaviour intention, often identified as the main determinant influencing purchase intention^4,30–32^. For those wishing to promote QR code pork purchase in the UK, it is therefore recommended that interventions are designed with consideration for consumers’ perceptions and attitudes, thus, enabling a greater recognition and appreciation for the value of this product. Although contributing comparatively less, PBC and personal concern towards AMU were also still significant drivers of intention in the extended model for the farm assurance label sub-group, however, knowledge of EU regulations was no longer a predictor. In the extended model for the antibiotic usage label sub-group, PBC, acceptance of animal AMU, perceptions towards animal welfare standards, and awareness of AMR were all still significant drivers of intention. Although meta-analysis^31^ has shown that the TPB variables have medium to large associations with both intention and behaviour, this study demonstrates that they have a lesser influence. While attitude was a strong precedent of purchase intention across both labelling sub-groups, PBC contributed comparatively less. In addition, trust did not emerge as a significant predictor of intention in either label sub-group. This finding was somewhat unexpected as not only is trust a component of the TPB, but it was expected to be an important factor as people rely on trust if they do not have much knowledge^2^; and our study determined that consumers indeed lack knowledge and awareness of AMR. Additionally, throughout the literature trust has been linked with consumer intention, found to both positively^3,4,32,57,58^ and negatively^59^ influence purchase intentions. Trust in the traceability system can persuade or dissuade consumers from purchasing products such as QR code labelled pork, as Menozzi et al.^32^ reported that consumer trust in the effectiveness of this system was the main determinant of intention to buy traceable chicken and honey. Therefore, future research should focus on building consumer trust in both antibiotic-related information and in the traceability system itself. It is also worth considering the source that might act as an avenue for consumer education and related information (i.e., government led campaigns), by exploring the trust placed in various stakeholders and organisations, it will be possible to identify the most effective manner in which to gain consumer trust and thus, advocate QR code labelled pork.

The use of antibiotic credence labelling such as “antibiotic-free”, “no antibiotics ever”, and “raised without antibiotics” has been used in abundance on food labels in countries such as the United States^60^; however, in recent years we have seen the emergence of a “raised without antibiotics” (RWA) label on the UK market. Despite this, the impact that RWA livestock production has on welfare parameters has not yet been quantified, and thus, it is possible that the elimination of antibiotics from production can have detrimental influence on animal welfare; particularly if sick animals are denied treatment in order to comply with marketing standards. Hence, there is a gap in the UK market for a product that provides valuable and useful antibiotic-related information while preserving animal welfare. QR code labelled pork may offer an alternative solution to RWA labelling, providing consumers with the necessary information without inadvertently communicating that conventionally produced ‘unlabelled’ pork is harmful or unsafe. QR codes can be used as a means of access to information via food packaging and therefore, any information can be added or removed to provide consumers with the exact information they require, highlighting that QR codes are an effective and adaptable platform of information dissemination. For instance, the present study has identified that consumers have a high level of consideration for animal welfare standards, subsequently, information could be added to the QR code output to provide additional animal welfare data (i.e., adequate housing, nutrition, vaccination, farm management); and this may be worth exploring in the future. Additionally, by providing this information on food labels, it may also encourage producers to reassess their AMU practices and influence positive reform through the application of improved farm management and prevention strategies.

Overall, based on the findings presented in this study, the use of QR codes as a means of access to antibiotic information may be considered as a suitable and useful alternative to RWA and other associated ‘antibiotic-free’ labelling. This finding assists to inform marketers, retailers, and policymakers to aid the development of effective strategies to further engage consumers and to successfully identify a position for this product in the UK market. Whilst we have outlined the practicality of QR code labelled pork, it is, however, imperative for these stakeholders to conduct cost-benefit analysis prior to the launch of any such product. Additionally, having uncovered a lack of consumer knowledge surrounding agricultural AMU practices, it is necessary to develop targeted communication materials to address consumer concern and misinformation, educating the consumer on the positive role of access to antibiotics in upholding animal welfare standards.

Notwithstanding the contributions of this research, certain limitations remain that future research should seek to overcome. Firstly, behavioural intention is reported in this study rather than actual behaviour; however, intention does not necessarily translate into purchasing behaviour^10^. Additionally, there is also the possibility that consumers may not notice or choose not to scan the QR code. Despite this, research suggests that nearly 60% of shoppers use their mobile phones to search for coupons, and more than half of shoppers use apps when grocery shopping, suggesting that many consumers feel confident using their mobile phone to seek product information while shopping^61^. Nevertheless, it must be considered that consumers are faced with various other types of information, labels, and logos when shopping which influences their purchase intention, and this is something that this research has not addressed. It is therefore recommended that future studies investigate the in-store purchase of QR code labelled pork, for instance, through the application of an experimental auction. Conducting in-store research, using real money and real goods will thereby assure researchers that consumer responses are more closely linked to their actual purchasing behaviour. Additionally, various design aspects should be considered before a QR code is added to product packaging. For instance, ensuring the QR code is the correct size for the food product and that it can be easily seen by consumers, and providing a call-to-action text (i.e., ‘scan me’) and brief instructions for scanning food QR codes as not all consumers are familiar with doing so^20^. Lastly, as this research was the first of its kind to investigate consumers’ perceptions and intention to buy a hypothetical QR code labelled pork product, the results presented are preliminary and future studies should seek to replicate these findings to ensure that the QR code output presented to consumers is of utmost success.

Overall, this study demonstrates that consumers have somewhat favourable perceptions and attitudes towards QR code pork as an antibiotic traceability system, and that these are the main determinants having a positive influence on their purchase intention. QR code labelled pork may be a suitable and useful alternative to RWA labelling however, more research is needed to directly compare QR code labelled pork and antibiotic-free labelled products before marketers can develop strategies to promote this traceable product. In addition, communication practitioners should place an emphasis on developing communications to increase consumer knowledge and awareness of agricultural AMU and address any misinformation.

## Methods

### Data collection and participants

Using an online survey (see Supplementary Table 1), data were collected investigating various behavioural, psychological, and social factors relating to the purchase intention of QR code labelled pork, among a nationally representative sample of adults living in the UK (male and female, aged 18–92). Individuals were invited to participate in the survey by a research agency (Dynata) from their online panel of UK consumers in May 2020. Individuals were paid a small fee to complete the survey. Respondents completed a series of screening questions to assess their eligibility to take part in the study. To avoid bias, anyone aged under 18 or working in the media, food safety, food processing, or farming/agriculture were excluded. Additionally, those who had no shopping responsibility, and purchased and consumed pork less than every few months were also excluded. Quotas were applied to achieve a nationally representative UK sample in terms of age, region, sex, and SES.

Respondents were randomly assigned to a survey with approximately half of the respondents (*n* = 495) answering questions related to QR code one (antibiotic usage labelled pork), and the other half (*n* = 505) answering questions related to QR code two (farm assurance labelled pork). To ensure no missing data, a forced response option was used for all items. The questionnaire took approximately 20 min to complete. Ethical approval for the study was obtained by the School of Medicine, Dentistry, and Biomedical Sciences Faculty Research Ethics Committee, Queen’s University Belfast (Faculty REC Reference Number: MHLS 20_23) and conducted in accordance with guidelines specified in the Declaration of Helsinki. Participants were told that by agreeing to take part in the survey they were providing consent and no written consent was obtained. The data was collected by a research agency, Dynata. An overview of participants socio-demographic characteristics is described in Table 1.

### Questionnaire design

The questionnaire contained close-ended questions pertaining to various theoretical constructs from the TPB^27^ and was initially piloted among eight individuals to assess practicality (i.e., structure, content, instructions, duration). Firstly, pork consumption, purchase frequency, and the importance of selected attributes in purchase decisions, were measured. Socio-demographic characteristics were then sought to ensure nationally representative quotas were obtained. Then, consumer knowledge of EU regulations, awareness of AMR, and AMU practices within both humans and animals, were measured. Following this, participants were presented with an example of a hypothetical QR code labelled pork product before completing items measuring attitude, PBC, trust, behavioural beliefs, perceptions of QR code, and purchase intention. Lastly, WTP, generalised trust, and remaining socio-demographics (education, marital status, occupation status, household income, number of children and adults in household) were recorded.

### Pictorial example of QR code labelled pork

Respondents were shown an example of a pork product with a QR code facilitating access to antibiotic-related information. The pictorial highlighted the difference to traditional pork widely available in stores due to the unique information made available to consumers by scanning the QR code on the pack via a smartphone. A visual aid showing QR code labelled pork was shown to respondents as illustrated in Fig. 1. Respondents were randomly assigned to a pork product with a QR code. Half of the respondents (*n* = 495) were shown QR code 1 (antibiotic usage labelled pork), and the other half (*n* = 505) were shown QR code 2 (farm assurance labelled pork).

The basis of each QR code option was the same. It enabled consumers to gain information about the region of origin, the farmer, the rearing conditions of the pork (e.g., indoor/outdoor), the breed of pig, and an assurance that the product is compliant with UK law, appropriate withdrawal periods, and RSPCA animal welfare standards. In addition, QR code 1 (referred to as the ‘antibiotic usage label’) provided quantified antibiotic usage data from the farm in mg/kg, while QR code 2 (referred to as the ‘farm assurance label’) said that the product is Red Tractor assured.

### Measures

All items were scored on a 7-point Likert-type scale (1 = “strongly disagree”, 7 = “strongly agree”, unless otherwise indicated). All items were adapted from Spence et al.^4^ unless otherwise indicated.

#### Attitude

Attitude towards purchasing QR code pork in comparison to traditional pork currently available in supermarkets was measured with four items on seven-point semantic differential scale. Participants were asked to indicate how purchasing pork labelled with antibiotic information would make them feel (foolish-wise, displeased-pleased) and beliefs towards purchase (foolish-wise, harmful-beneficial).

#### Perceived behavioural control

Respondent’s perceived ability to find and understand the antibiotic information embedded in the QR code label was assessed by eight items.

#### Trust

Trust in the QR code antibiotic information was evaluated with three items: “I trust that QR code labelled pork can provide accurate and reliable information surrounding antibiotic use during production”, “I trust that the information about adherence to the withdrawal period is reliable on QR code labelled pork” and “I trust that QR code labelled pork will provide an assurance that antibiotics have been used on the animal responsibly”.

#### Behavioural beliefs

To measure behavioural beliefs, eight statements that compared QR code labelled pork to traditional pork currently available in supermarkets were measured (e.g., QR code labelled pork will likely be: healthier, more expensive, tastier, easier to find, of more satisfying quality, safer to eat, have higher animal welfare standards, be free from antibiotics).

#### Perceptions of QR code

Respondent’s perceptions towards QR code pork were measured with seven items (constructed by the author): “I believe this QR code would be useful”, “I would like to see this QR code on pork products”, “seeing this QR code on foods will assure me that antibiotics have been used on the animal responsibly”, “I would eat meat from animals which had antibiotics knowing that the animal hasn’t suffered”, “buying products with this QR code will reduce my risk of consuming antibiotics”, “buying products with this QR code will reduce my chances of getting AMR” and “buying products with this QR code will help me not worry as much about AMR”.

#### Purchase intention

To measure intention to purchase QR code labelled pork, participants responded to four statements: “if pork products with this QR code become available…”, “I intend to buy them”, “I will look for them”, “it will be important for me to buy them” and “I will buy them to find out more about animal welfare standards”.

#### Generalised trust

Respondents rated the extent to which they trust others with four items (unpublished): “most people are basically honest”, “most people are trustworthy”, “most people are basically good and kind” and “most people are trustful of others”.

#### Perception of AMU practices

Perceptions towards human and animal AMU practices were assessed by eleven items (adapted from Goddard et al.^11^); three relating to human practices and eight in relation to animal practices.

#### Pork purchasing habits

To measure the importance of pork attributes, respondents were shown thirteen items to identify the factors most important to them when purchasing pork products (e.g., price, quality, quantity, appearance, origin, antibiotics used, organic, animal welfare practices, place of purchase, the brand, healthiness, environmental friendliness, type of packaging).

#### WTP

Respondents indicated how much more (in pence) they would pay for QR code labelled pork directly through the question: “suppose the price of pork currently available in the supermarket is £2 for 500 g. The price of pork labelled with a QR code embedded with unique antibiotic information will be higher but is not determined yet. How much more would you be willing to pay to purchase 500 g of labelled pork?” Participants chose between the following options: 10p, 20p, 30p, 40p, 50p, 60p, 70p, 80p, 90p, £1, £1.10, £1.20, £1.30, £1.40, £1.50 + , “I would not be willing to pay any extra” and “I would not be willing to buy pork labelled with a QR code”. The price of the traditional pork was based upon the market price of a product sold in September 2019 by one leading UK supermarket.

#### Knowledge of EU regulations

Respondent’s knowledge of EU regulated pork was evaluated by five items (constructed by the author) and measured using a dichotomous scale (true/false).

#### Awareness of AMR

Awareness towards AMR was assessed by two dichotomous scale (yes/no) items (adapted from Goddard et al.^11^): “have you heard of antibiotic or antimicrobial resistance?” and “do you know what AMR is?”

### Data analysis

All data were analysed using IBM SPSS Statistics version 26.0 (IBM Corporation, Armonk, NY, USA), with a p-value *p* ≤ 0.05 considered to be significant.

#### Construction of variable scales

Each of the 62 Likert-type questionnaire items were entered into a maximum likelihood factor analysis with Direct Oblimin rotation, supressing factor loadings < 0.3, to identify a measurement model. The item loadings were then examined to ensure that a clean solution was attained and Cronbach’s α coefficient was calculated to investigate the internal reliability of each construct (Table 2), with an α value of > 0.70 deemed acceptable^62^. The items within each construct were then averaged by computing a mean of the loading items and scales were constructed. Scores of each scale ranged from a minimum of 1 to a maximum of 7, with higher values signifying stronger levels of the construct. Finally, resulting scales were labelled based upon their content and Pearson correlations were computed to measure the strength of the relationship between constructs within the model.

#### Descriptive analysis

Descriptive statistics (mean and SD) were used to explore the data within each sub-group (Table 2).

#### Regression analysis

Hierarchical multiple regression analyses were used to examine the association between predictor variables and intention to purchase QR code labelled pork across both sub-groups. In step 1, exploratory predictor variables were entered while socio-demographic characteristics were entered in step 2. In addition, to examine the unique contribution of behavioural beliefs scores and attitude, a standard multiple regression analysis was performed. Regression assumptions regarding normality, linearity, and homoscedasticity were met and multicollinearity was not a concern (all correlation coefficients were < 0.80 and all tolerance statistics were > 0.2).

### Factor analysis

Perceptions of QR code (α = 0.92), intention (α = 0.95), attitude (α = 0.93), trust (α = 0.94), perceived behavioural control (α = 0.93), and generalised trust (α = 0.93) were all unifactorial. Behavioural beliefs also yielded a one-factor solution however, for structure and practicality, beliefs were separated into three groups. Group one consisted of four attributes relating to the quality of QR code labelled pork (and was therefore labelled *Quality*, α = 0.88), group two consisted of three attributes relating to animal welfare practices involved in the production of QR code labelled pork (and was therefore labelled *Animal welfare*, α = 0.83, and group three consisted of one attribute relating to the cost of the product (and was therefore labelled *Expense*). Perceptions of AMU practices contained four factors, identified as *Personal concern* (α = 0.65), *Animal welfare standards* (*p* = 0.75), *Animal AMU acceptance* (α = 0.71), and *Animal concern* (α = 0.55). Due to unacceptable internal reliability, the factor Animal concern was removed from the analysis. Although the internal reliability of Personal concern was also < 0.7 (α = 0.65), it was deemed acceptable for analysis as recommended by Ursachi et al.^63^ as the α is within the range of 0.6–0.7. All factor items and internal reliability values are presented in Table 2.

## Supplementary information

Consumer survey

Published paper sharing data

## Data Availability

The authors declare that the data supporting the findings presented in this study are available within the paper and raw data can be accessed at https://osf.io/b5c63/quickfiles.
