# Supplementary material for: Consumer purchase intention towards a quick response (QR) code for antibiotic information: an exploratory study
Source: NPJ Sci Food. 2022 Apr 20;6:23. doi: 10.1038/s41538-022-00136-4 (PMC9021225; doi:10.1038/s41538-022-00136-4)
Supplement: Supplementary file 1 — Consumer survey [file 41538_2022_136_MOESM1_ESM.pdf]

Supplementary Information

Consumer purchase intention towards a Quick Response (QR) code for antibiotic information: An exploratory study

Hollie Bradford*, Claire McKernan, Chris Elliott, and Moira Dean

Institute for Global Food Security, School of Biological Sciences, Queen's University Belfast, 19 Chlorine Gardens, Belfast BT9 5DL, United Kingdom

*To whom correspondence should be addressed: Email: hbradford01@qub.ac.uk

Supplementary Table 1. Online consumer survey.

<table>
<tr><th>Variable type</th><th>Source / notes</th><th>Questionnaire items</th></tr>
<tr><td colspan="3">INTRODUCTION

The following survey is on food and aims to understand the choices that consumers make when purchasing pork. We are looking for specific people to take part in the survey and so we need to ask you a few selection questions before you are invited into the main study, which would take around 20 – 25 minutes to complete.

All information collected during the survey will be kept strictly confidential. The data will be processed anonymously and you will not be personally identifiable in any research outputs or reports. As data is anonymised you will be unable to withdraw your data however, you may leave the study at any time by closing your browser window. Please note that by taking part in this survey you are providing consent for your data to be included in this research.

You should answer all questions as openly and honestly as possible. Questions are based on attitudes and perceptions, there are no right and wrong answers. At the end of the survey you will be required to provide some general demographic information.

This project is a DfE research studentship.</td></tr>
<tr><td colspan="3">SCREENING QUESTIONS</td></tr>
<tr><td>SHOPPING RESPONSIBILITY</td><td>Participants must have at least some purchasing responsibility in order to answer questions surrounding their intentions to buy pork labelled with antibiotic information.</td><td>QS1. Are you responsible for the food and grocery shopping in your household?
<table><tr><td>No – someone else does it</td><td>1</td><td>CLOSE</td></tr><tr><td>Yes – I am jointly responsible / share responsibility with others</td><td>2</td><td></td></tr><tr><td>Yes – I do most of the food and grocery shopping</td><td>3</td><td></td></tr></table></td></tr>
</table>

<table>
<tr>
<td>PORK PURCHASING</td>
<td>In order for individuals to answer questions on factors important to them when purchasing pork, they will need to purchase some pork products themselves.</td>
<td>QS2. Approximately how often do you purchase pork from the supermarket? This includes every type of pork and pork products that may be purchased on their own or as part of a meal.
<table>
<tr><td>Never</td><td>1</td><td>CLOSE</td></tr>
<tr><td>Less than once a year</td><td>2</td><td>CLOSE</td></tr>
<tr><td>Once or twice a year</td><td>3</td><td>CLOSE</td></tr>
<tr><td>Every few months</td><td>4</td><td></td></tr>
<tr><td>1-4 times a month</td><td>5</td><td></td></tr>
<tr><td>A few times a week</td><td>6</td><td></td></tr>
<tr><td>Daily</td><td>7</td><td></td></tr>
</table>
</td>
</tr>
<tr>
<td>PORK CONSUMPTION</td>
<td>In order for individuals to answer questions on factors important to them when purchasing pork and use of antibiotics in livestock, they will need to consume at least one pork product.</td>
<td>QS3. How often do you eat pork bought from the supermarket? This includes every type of pork and pork products that may be purchased on their own or as part of a meal (sausages, pepperoni, ham, bacon, pulled pork, sausage rolls, pork pies etc.)
<table>
<tr><td>Never</td><td>1</td><td>CLOSE</td></tr>
<tr><td>Less than once a year</td><td>2</td><td>CLOSE</td></tr>
<tr><td>Once or twice a year</td><td>3</td><td>CLOSE</td></tr>
<tr><td>Every few months</td><td>4</td><td></td></tr>
<tr><td>1-4 times a month</td><td>5</td><td></td></tr>
<tr><td>A few times a week</td><td>6</td><td></td></tr>
<tr><td>Daily</td><td>7</td><td></td></tr>
</table>
</td>
</tr>
</table>

<table>
<tr><td>AGE</td><td>Participants must be 18+</td><td>QS4. What is your age? (Insert age)

CLOSE FOR INDIVIDUALS UNDER 18</td></tr>
<tr><td>GENDER</td><td></td><td>QS5. I identify my gender as:
<table>
<tr><td>Male</td><td>1</td></tr>
<tr><td>Female</td><td>2</td></tr>
<tr><td>Other</td><td>3</td></tr>
</table></td></tr>
<tr><td>OCCUPATION</td><td>Participant cannot work in the media or agriculture/ food sector.</td><td>QS6. Do you work in any of the following occupations?
<table>
<tr><td>Teaching</td><td>1</td><td></td></tr>
<tr><td>Banking/finance</td><td>2</td><td></td></tr>
<tr><td>The media</td><td>3</td><td>CLOSE</td></tr>
<tr><td>Food safety</td><td>4</td><td>CLOSE</td></tr>
<tr><td>Food processing</td><td>5</td><td>CLOSE</td></tr>
<tr><td>Farming/agriculture</td><td>6</td><td>CLOSE</td></tr>
<tr><td>None of these</td><td>7</td><td></td></tr>
</table></td></tr>
<tr><td>SEG</td><td></td><td>QS7. Which one of the following categories best describes the employment of the main income earner in your household?
<table>
<tr><td>Work as a professional such as a lawyer, doctor, accountant or other occupation that requires extensive education and training</td><td>1</td></tr>
<tr><td>Very senior manager in a business or commerce</td><td>2</td></tr>
</table></td></tr>
</table>

		Top-level civil servant	3
		Self-employed with 25+ employees	4
		Self-employed with 5-24 employees	5
		Self-employed with 1-4 employees (in addition to you)	6
		Middle management executive in large organisation	7
		Junior management	8
		Principal officer in local government or civil service	9
		Other public services (police, health, education, fire service)	10
		Other non-manual work, such as clerical, secretarial, or sales	11
		Skilled manual worker (working in a trade or craft occupation)	12
		Other manual worker with responsibility for other people	13
		Semi-skilled worker or worker in manual or service occupation	14
		Apprentice or trainee to skilled worker	15
		Student	16
		Still at school	17
		Unemployed and seeking work	18
		Retired	19
		Not in paid work for other reason	20
		Never worked	21

SECTION A: PORK PURCHASING HABITS		
IMPORTANCE OF PORK ATTRIBUTES	This will enable identification of the factors most important to consumers when purchasing pork products.	Q1. Please rate the following based on their level of importance when purchasing pork:

	Extremely unimportant	Unimportant	Somewhat unimportant	Important nor unimportant	Somewhat important	Important	Extremely important
Price	1	2	3	4	5	6	7
Quality (for example, taste/flavour/ freshness)	1	2	3	4	5	6	7
Quantity (for example, size)	1	2	3	4	5	6	7
Appearance (for example, colour/texture)	1	2	3	4	5	6	7
Origin (for example, local, British, EU)	1	2	3	4	5	6	7
Antibiotics used	1	2	3	4	5	6	7
Organic (or other assurance certificate)	1	2	3	4	5	6	7
Animal welfare practices	1	2	3	4	5	6	7

Place of purchase	1	2	3	4	5	6	7
The brand	1	2	3	4	5	6	7
Healthiness/ nutritional content	1	2	3	4	5	6	7
Environmental friendliness	1	2	3	4	5	6	7
The type of packaging	1	2	3	4	5	6	7

SECTION B: KNOWLEDGE AND AWARENESS OF CURRENT EU REGULATED PORK PRODUCTS

KNOWLEDGE OF CURRENT EU REGULATED PORK PRODUCTS	These questions will explore consumer knowledge and awareness of pork products sold in the EU and explore their perceptions towards these products. Questions will include awareness towards EU regulation and laws. Participants will be provided with information relating to these questions at the end of the survey in the participant debrief.	Q2. Please read the following statements and indicate if you think they are true or false.

	True	False
All food products sold in the UK are regulated under European Union (EU) law to ensure that they are safe for human consumption	1	2
Growth promoters are often used to stimulate rapid weight gain in food producing animals sold in the UK	1	2
There are no regulations or restrictive provisions on antibiotics used in food producing animals in the UK	1	2
If food producing animals are treated with antibiotics then antibiotics will be present in the meat	1	2
All food produced and sold in the UK complies with packaging, labelling and safety requirements	1	2

SECTION C: AWARENESS OF ANTIBIOTIC USE AND RESISTANCE		
AWARENESS OF AMR	General questions to assess participant's awareness of AMR.	Q3. Have you ever taken antibiotics before in your lifetime?<br><br>No: 1<br>Yes: 2<br><br>***(If answered 'no', skip to Q6)**<br><br>Q4. Have you taken antibiotics in the last 12 months?<br><br>No: 1<br>Yes: 2<br><br>Q5. Have you had a situation where antibiotics didn't work? (*This means you were told by a medical professional that the antibiotic didn't work*)<br><br>No: 1<br>Yes: 2<br><br>Q6. Have you heard of antibiotic or antimicrobial resistance (AMR)?<br><br>No: 1<br>Yes: 2

		Q7. Do you know what AMR is?<br><br>No – 1<br>Yes – 2<br><br>AFTER ANSWER, PROVIDE THE DEFINITION "AMR is the ability of micro-organisms to withstand antimicrobial treatment such as antibiotics. This means that antibiotics may not work, making it difficult or impossible to treat illnesses and infections."
**SECTION D: PERCEPTION OF AMU PRACTICES**		
PERSONAL ANTIBIOTIC USE<br><br>ANTIBIOTIC USE IN LIVESTOCK	Questions on personal antibiotic use and use on animals.	Q8. To what extent do you agree or disagree with each of the following statements? Please use a scale of 1 to 7, where 1 means strongly disagree and 7 means strongly agree.

No	1
Yes	2

	Strongly disagree						Strongly agree
I am concerned that AMR will affect me one day	1	2	3	4	5	6	7
Too many antibiotics from the doctor can cause AMR	1	2	3	4	5	6	7
If I have AMR I will not be able to treat illness	1	2	3	4	5	6	7

It is important to me that animal welfare standards are adhered to when purchasing meat	1	2	3	4	5	6	7
It is important to me that the pork I buy has been produced in a way that the animal has experienced as little pain as possible	1	2	3	4	5	6	7

		I would be willing to consume meat from animals treated with antibiotics	1	2	3	4	5	6	7
		Using antibiotics in livestock makes them less effective in humans	1	2	3	4	5	6	7
		Overall, the use of animal antibiotics delivers more benefits than harm	1	2	3	4	5	6	7
		The use of antibiotics in livestock cannot be seriously harmful, otherwise usage would be banned	1	2	3	4	5	6	7
		Antibiotics should never be used in livestock production, even in medical need, since it is critical to maintain useful antibiotics for public health use	1	2	3	4	5	6	7
		I consider domestic pets to be a potential source of transfer of AMR	1	2	3	4	5	6	7

SECTION E: PERCEPTIONS TOWARDS PORK LABELLED WITH ANTIBIOTIC RELATED INFORMATION		
BEHAVIOURAL BELIEFS TOWARDS QR CODE LABELLED PORK	Questions to explore consumer's beliefs, attitudes and purchase intentions towards antibiotic usage information on packaging (QR code).	You will now see an example of a pork product with a QR code which will give you information about antibiotic usage. 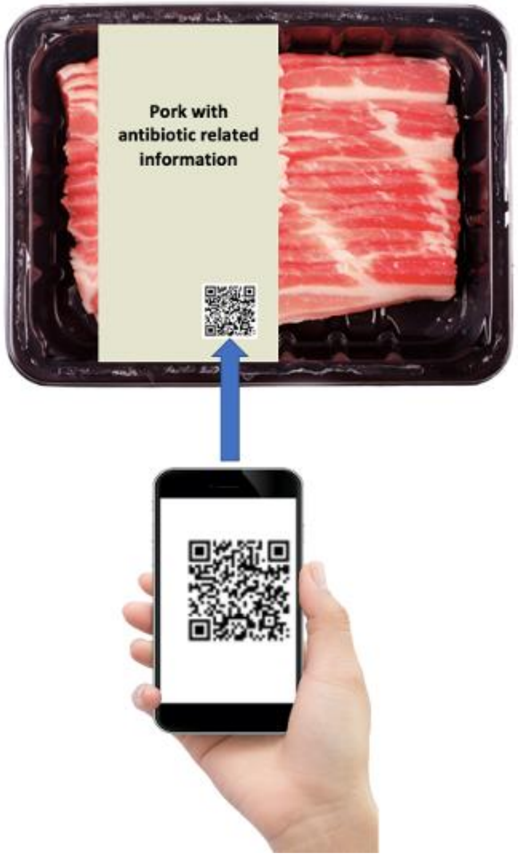

<table>
<tr>
<td></td>
<td></td>
<td>

OPTION 1 (500 PARTICIPANTS ONLY)

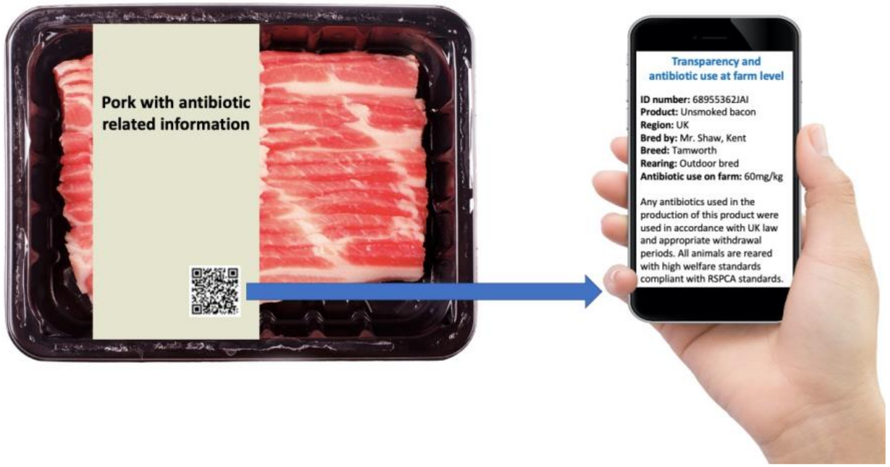

OPTION 2 (REMAINING 500 PARTICIPANTS ONLY)

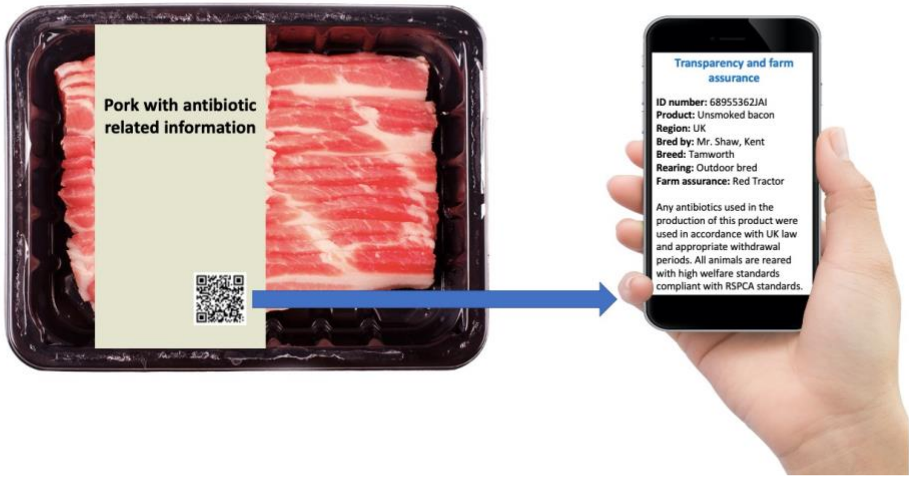

</td>
</tr>
</table>

<table>
<tr>
<td></td>
<td>Questions being asked in this section about QR code labelled pork are in comparison to the traditional product.</td>
<td>Q9. Based on the idea of QR code labelled pork becoming available, in comparison to traditional pork currently available in supermarkets, to what extent do you agree or disagree with each of the following statements? Please use a scale of 1 to 7, where 1 means strongly disagree and 7 means strongly agree.
<table>
<tr><td></td><td>Strongly disagree</td><td></td><td></td><td></td><td></td><td>Strongly agree</td></tr>
<tr><td>QR code labelled pork will likely be healthier</td><td>1</td><td>2</td><td>3</td><td>4</td><td>5</td><td>6</td><td>7</td></tr>
<tr><td>QR code labelled pork will likely be more expensive</td><td>1</td><td>2</td><td>3</td><td>4</td><td>5</td><td>6</td><td>7</td></tr>
<tr><td>QR code labelled pork will likely be tastier</td><td>1</td><td>2</td><td>3</td><td>4</td><td>5</td><td>6</td><td>7</td></tr>
<tr><td>QR code labelled pork will likely be easier to find</td><td>1</td><td>2</td><td>3</td><td>4</td><td>5</td><td>6</td><td>7</td></tr>
<tr><td>QR code labelled pork will likely be of more satisfying quality</td><td>1</td><td>2</td><td>3</td><td>4</td><td>5</td><td>6</td><td>7</td></tr>
<tr><td>QR code labelled pork will likely be safer to eat</td><td>1</td><td>2</td><td>3</td><td>4</td><td>5</td><td>6</td><td>7</td></tr>
<tr><td>QR code labelled pork will likely have higher animal welfare standards</td><td>1</td><td>2</td><td>3</td><td>4</td><td>5</td><td>6</td><td>7</td></tr>
<tr><td>QR code labelled pork will likely be free from antibiotics</td><td>1</td><td>2</td><td>3</td><td>4</td><td>5</td><td>6</td><td>7</td></tr>
<tr><td>I would be more willing to buy pork products with this QR code than those without</td><td>1</td><td>2</td><td>3</td><td>4</td><td>5</td><td>6</td><td>7</td></tr>
<tr><td>I would be willing to pay more for this pork product than a</td><td>1</td><td>2</td><td>3</td><td>4</td><td>5</td><td>6</td><td>7</td></tr>
</table>
</td>
</tr>
</table>

<table>
<tr><td></td><td></td><td><table><tr><td>traditional pork product without the QR code</td><td></td><td></td><td></td><td></td><td></td><td></td><td></td></tr></table></td></tr>
<tr><td>PERCEPTIONS OF QR CODE</td><td></td><td>Q10. Based on the idea of QR code labelled pork becoming available, to what extent do you agree or disagree with each of the following statements? Please use a scale of 1 to 7, where 1 means strongly disagree and 7 means strongly agree.<table>
<tr><td></td><td>Strongly disagree</td><td></td><td></td><td></td><td></td><td></td><td>Strongly agree</td></tr>
<tr><td>I believe this QR code would be useful</td><td>1</td><td>2</td><td>3</td><td>4</td><td>5</td><td>6</td><td>7</td></tr>
<tr><td>I would like to see this QR code on pork products</td><td>1</td><td>2</td><td>3</td><td>4</td><td>5</td><td>6</td><td>7</td></tr>
<tr><td>Seeing this QR code on foods will assure me that antibiotics have been used on the animal responsibly</td><td>1</td><td>2</td><td>3</td><td>4</td><td>5</td><td>6</td><td>7</td></tr>
<tr><td>I would eat meat from animals which had antibiotics knowing that the animal hasn’t suffered</td><td>1</td><td>2</td><td>3</td><td>4</td><td>5</td><td>6</td><td>7</td></tr>
<tr><td>Buying products with this QR code will reduce my risk of consuming antibiotics</td><td>1</td><td>2</td><td>3</td><td>4</td><td>5</td><td>6</td><td>7</td></tr>
<tr><td>Buying products with this QR code will reduce my chances of getting AMR</td><td>1</td><td>2</td><td>3</td><td>4</td><td>5</td><td>6</td><td>7</td></tr>
<tr><td>Buying products with this QR code will help me not worry as much about AMR</td><td>1</td><td>2</td><td>3</td><td>4</td><td>5</td><td>6</td><td>7</td></tr>
</table></td></tr>
</table>

<table>
<tr>
<td>INTENTION TO BUY QR CODE LABELLED PORK</td>
<td></td>
<td>Q11. Use the scale from 1 to 7 to rate your disagreement or agreement with the following statements:
<table>
<tr><td></td><td>Strongly disagree</td><td></td><td></td><td></td><td></td><td>Strongly agree</td></tr>
<tr><td>If pork products with this QR code become available, I intend to buy them</td><td>1</td><td>2</td><td>3</td><td>4</td><td>5</td><td>6</td><td>7</td></tr>
<tr><td>If pork products with this QR code become available, I will look for them</td><td>1</td><td>2</td><td>3</td><td>4</td><td>5</td><td>6</td><td>7</td></tr>
<tr><td>If pork products with this QR code become available, it will be important for me to buy them</td><td>1</td><td>2</td><td>3</td><td>4</td><td>5</td><td>6</td><td>7</td></tr>
<tr><td>If pork products with this QR code become available, I will buy them to find out more about animal welfare standards</td><td>1</td><td>2</td><td>3</td><td>4</td><td>5</td><td>6</td><td>7</td></tr>
</table>
</td>
</tr>
<tr>
<td>WILLINGNESS TO PAY</td>
<td></td>
<td>Q12. Suppose the price of traditional pork currently available in the supermarket is £2 for 500g. The price of QR code labelled pork embedded with unique antibiotic information will be higher but is not determined yet. How much more would you be willing to pay to purchase 500g of labelled pork?
<table>
<tr><td>I would not be willing to buy QR code labelled pork</td><td>1</td></tr>
<tr><td>I would not be willing to pay any extra</td><td>2</td></tr>
<tr><td>10p (£2.10)</td><td>3</td></tr>
<tr><td>20p (£2.20)</td><td>4</td></tr>
<tr><td>30p (£2.30)</td><td>5</td></tr>
<tr><td>40p (£2.40)</td><td>6</td></tr>
</table>
</td>
</tr>
</table>

<table>
<tr>
<td></td>
<td></td>
<td>
<table>
<tr><td>50p (£2.50)</td><td>7</td></tr>
<tr><td>60p (£2.60)</td><td>8</td></tr>
<tr><td>70p (£2.70)</td><td>9</td></tr>
<tr><td>80p (£2.80)</td><td>10</td></tr>
<tr><td>90p (£2.90)</td><td>11</td></tr>
<tr><td>£1 (£3)</td><td>12</td></tr>
<tr><td>£1.10 (£3.10)</td><td>13</td></tr>
<tr><td>£1.20 (£3.20)</td><td>14</td></tr>
<tr><td>£1.30 (£3.30)</td><td>15</td></tr>
<tr><td>£1.40 (£3.40)</td><td>16</td></tr>
<tr><td>£1.50 + (£3.50+)</td><td>17</td></tr>
</table>
</td>
</tr>
<tr>
<td>ATTITUDES TOWARDS PURCHASING QR CODE LABELLED PORK</td>
<td></td>
<td>
Q13. Using a scale of 1 to 7, please rate the following statements using the words at the end of each scale. For example, for the first question, if you believe buying QR code labelled pork instead of traditional pork would be very bad you would select ‘1’, if you believe buying QR code labelled pork instead of traditional pork would be very good you would select ‘7’. An answer of ‘4’ for any statement indicates that you believe that there is no difference between QR code labelled pork and traditional pork for that statement.

Buying QR code labelled pork would make me feel…
<table>
<tr><td>Very bad</td><td></td><td></td><td>No difference</td><td></td><td></td><td>Very good</td></tr>
<tr><td>1</td><td>2</td><td>3</td><td>4</td><td>5</td><td>6</td><td>7</td></tr>
</table>
</td>
</tr>
</table>

Very displeased			No difference			Very pleased
1	2	3	4	5	6	7

I think that buying QR code labelled pork would be…

Very foolish			No difference			Very wise
1	2	3	4	5	6	7

Very harmful			No difference			Very beneficial
1	2	3	4	5	6	7

TRUST IN QR CODE LABELLED PRODUCTS

Q14. To what extent do you agree or disagree with each of the following statements?

	Strongly disagree						Strongly agree
I trust that QR code labelled pork can provide accurate and reliable information surrounding antibiotic use during production	1	2	3	4	5	6	7
I trust that the information about adherence to the withdrawal period is reliable on QR code labelled pork	1	2	3	4	5	6	7
I trust that QR code labelled pork will provide an assurance that antibiotics have been used on the animal responsibly	1	2	3	4	5	6	7

PERCEIVED BEHAVIOURAL CONTROL		Q15. Do you have a smartphone?

Q15. Do you have a smartphone?

No	1
Yes	2

***(If answered 'no', skip to Q24)**

Q16. (**If yes**) have you ever used your smartphone to scan a QR code?

No	1
Yes	2

Q17. Based on the idea of QR code labelled pork becoming available, to what extent do you agree or disagree with each of the following statements? Please use a scale of 1 to 7, where 1 means strongly disagree and 7 means strongly agree.

	Strongly disagree						Strongly agree
It will be easy to find the antibiotic information	1	2	3	4	5	6	7
I am confident that I'll find the antibiotic information	1	2	3	4	5	6	7
I will be able to find the antibiotic information without help from others	1	2	3	4	5	6	7
It will be easy to understand the antibiotic information	1	2	3	4	5	6	7
I am confident that I'll understand the antibiotic information	1	2	3	4	5	6	7

I will be able to understand the antibiotic information without help from others	1	2	3	4	5	6	7
I would prefer to see a rating system or colour coding to indicate if antibiotic use is high rather than a figure or statement (e.g. similar to the traffic light rating system)	1	2	3	4	5	6	7
Even if I don’t understand the information I can use this label as a form of assurance that antibiotics have been used responsibly (withdrawn & safe)	1	2	3	4	5	6	7

SECTION F: GENERALISED TRUST

GENERALISED TRUST

Q18. To what extent do you agree or disagree with each of the following statements?

	Strongly disagree						Strongly agree
Most people are basically honest	1	2	3	4	5	6	7
Most people are trustworthy	1	2	3	4	5	6	7
Most people are basically good and kind	1	2	3	4	5	6	7
Most people are trustful of others	1	2	3	4	5	6	7

SECTION G: DEMOGRAPHICS AND PSCYHOSOCIAL FACTORS		
MARITAL STATUS		QD1. What is your marital status?
HIGHEST EDUCATION		QD2. What is the highest level of education that you have completed?

QD1. What is your marital status?

Married	1
Single (never married)	2
Widowed	3
Divorced	4
Separated	5
Living with partner	6

QD2. What is the highest level of education that you have completed?

None	0
Primary education completed	1
Secondary education completed (GCSE/O-Level/CSE or equivalent)	2
Secondary education completed (A-Level or equivalent)	3
Vocational or technical qualifications completed (e.g. HND, NVQ)	4
University education completed (first degree e.g. BA, BSc)	5
Postgraduate education completed (e.g. Masters)	6
Doctorate, post-doctorate or equivalent (Higher degree)	7

<table>
<tr>
<td>OCCUPATION STATUS</td>
<td></td>
<td>QD3. What is your work status?
<table>
<tr><td>Employed full time (>30 hours per week)</td><td>1</td></tr>
<tr><td>Employed part time (<29 hours per week)</td><td>2</td></tr>
<tr><td>Full time homemaker</td><td>3</td></tr>
<tr><td>Unemployed</td><td>4</td></tr>
<tr><td>Student</td><td>5</td></tr>
<tr><td>Retired</td><td>6</td></tr>
</table>
</td>
</tr>
<tr>
<td>HOUSEHOLD INCOME</td>
<td></td>
<td>QD4. What is the <u>total income of your household</u> from all sources before any tax and national insurance contributions? If you share your household with individuals unrelated to you (not a family member or your partner), please count only your personal income. INCLUDE ALL INCOME FROM EMPLOYMENT AND BENEFITS.
<table>
<tr><td>Under £6,999 per annum (less than £135 per week)</td><td>1</td></tr>
<tr><td>£7,000 - £9,999 per annum (£135 - £195 per week)</td><td>2</td></tr>
<tr><td>£10,000 - £14,999 per annum (£195 - £290 per week)</td><td>3</td></tr>
<tr><td>£15,000 - £19,999 per annum (£290 - £385 per week)</td><td>4</td></tr>
<tr><td>£20,000 - £25,999 per annum (£385 - £500 per week)</td><td>5</td></tr>
<tr><td>£26,000 - £29,999 per annum (£500 - £580 per week)</td><td>6</td></tr>
<tr><td>£30,000 - £39,999 per annum (£580 - £770 per week)</td><td>7</td></tr>
<tr><td>£40,000 - £49,999 per annum (£770 - £960 per week)</td><td>8</td></tr>
<tr><td>£50,000 - £59,999 per annum (£960 - £1,150 per week)</td><td>9</td></tr>
<tr><td>£60,000 + per annum (£1,150 per week)</td><td>10</td></tr>
</table>
</td>
</tr>
</table>

		Not sure — 11<br>Prefer not to say — 12
HOUSEHOLD SIZE / COMPOSITION		QD5. How many children aged under 16 live in your household?<br>[ ]<br>QD6. Including you, how many adults aged 16 or over live in your household?<br>[ ]
