# Supplementary material for: Consumer purchase intention towards a quick response (QR) code for antibiotic information: an exploratory study
Source: NPJ Sci Food. 2022 Apr 20;6:23. doi: 10.1038/s41538-022-00136-4 (PMC9021225; doi:10.1038/s41538-022-00136-4)
Supplement: Supplementary file 2 — Published paper sharing data [file 41538_2022_136_MOESM2_ESM.pdf]

Appetite 171 (2022) 105900

Contents lists available at ScienceDirect

Appetite

journal homepage: www.elsevier.com/locate/appet

Consumers' perceptions and willingness to purchase pork labelled 'raised without antibiotics'

Hollie Bradford [*], Claire McKernan, Chris Elliott, Moira Dean

Institute for Global Food Security, School of Biological Sciences, Queen's University Belfast, 19 Chlorine Gardens, Belfast, BT9 5DL, United Kingdom

ARTICLE INFO

Keywords:
Consumer perception
Antimicrobial resistance
Antibiotic free
Raised without antibiotics
Food label
Pork production

ABSTRACT

As a recent addition to the UK market, it is unknown how 'raised without antibiotics' labelled products are perceived or how they influence consumer food choice. Understanding consumers' perceptions towards the 'raised without antibiotics' label can determine knowledge of the label and what drives them to purchase products raised without antibiotics. Subsequently, using an online questionnaire with 1000 participants aged 18–92 years old, this study explored UK consumers' perceptions and willingness to buy 'raised without antibiotics' labelled pork, and examined their knowledge of antimicrobial use and antimicrobial resistance (AMR[1]). Cross-sectional data were collected investigating consumer perceptions and willingness to purchase 'raised without antibiotics' labelled pork and hierarchical multiple regression analyses were conducted. Respondents had high levels of knowledge towards EU regulations however, their awareness of AMR was limited. Behavioural beliefs concerning animal welfare and product quality were the main predictors of consumers' willingness to buy 'raised without antibiotics' labelled pork, followed by pork purchasing habits based on animal welfare qualities. Additionally, consumers who were more concerned that AMR would personally affect them, as well as those who favoured antimicrobial use for the preservation of animal welfare were willing to buy this product. Perceptions towards animal antimicrobial use acceptance, beliefs that 'raised without antibiotics' labelled pork is expensive, and a lack of consideration for extrinsic qualities when purchasing pork i.e., appearance, had a negative influence on willingness to buy. Moderate perceptions towards 'raised without antibiotics' labelled pork, lack of knowledge of agricultural antimicrobial use, and the subsequent confusion surrounding this label suggests that it is necessary to explore other labelling options to provide consumers with the information they desire while also safeguarding animal welfare.

1. Introduction

Antimicrobial resistance (AMR) is a worldwide public health concern diminishing the effectiveness of medication to treat illness with serious consequences for both animal and public health (Bokma et al., 2018). As awareness of AMR has increased over the years, concerns have arisen surrounding antimicrobial use in food-producing animals, particularly towards the potential zoonotic transfer of resistant bacteria from animals to humans (Ekakoro et al., 2019). This cross-species transmission is thought to occur through direct contact between humans and animals and indirectly via the environment and food chain (Sadiq et al., 2018); however, there is currently no robust evidence to demonstrate the extent or quantify the level of risk to humans from animal antimicrobial use (Coyne et al., 2018; Ekakoro et al., 2019). Within the UK, the pig sector has been identified as an area of particular concern, contributing to the greatest amount of antimicrobial sales across all livestock sectors (Coyne et al., 2014). Consequently, antimicrobial use is one of the most criticised issues in modern pig production (Grunert et al., 2018); resulting in proposals to reduce or entirely eliminate antimicrobial use in livestock production (Lusk et al., 2006).

Changing consumer preferences towards food safety has led to increasing interest in credence attributes (i.e., attributes that cannot be evaluated prior to purchase or consumption) (Grunert et al., 2018) and the proliferation of food labels such as 'raised without antibiotics' in an effort to differentiate in the pork market (McCarthy et al., 2004; Karavolias et al., 2018). The use of credence labelling as a marketing

* Corresponding author. 19 Chlorine Gardens, Belfast, BT9 5DL, Northern Ireland, United Kingdom.
E-mail addresses: hbradford01@qub.ac.uk (H. Bradford), c.mckernan@qub.ac.uk (C. McKernan), chris.elliott@qub.ac.uk (C. Elliott), moira.dean@qub.ac.uk (M. Dean).

[1] Antimicrobial resistance.

https://doi.org/10.1016/j.appet.2021.105900
Received 20 July 2021; Received in revised form 1 November 2021; Accepted 23 December 2021
Available online 27 December 2021
0195-6663/Crown Copyright © 2022 Published by Elsevier Ltd. This is an open access article under the CC BY license (http://creativecommons.org/licenses/by/4.0/).

technique is used within the pork industry, influencing consumers to consider aspects of livestock production when choosing products and capturing a higher price for 'niche' pork that has been produced with concern for ethical attributes such as animal welfare (Abrams et al., 2010; Grunert et al., 2018). The rapid growth of niche markets (i.e., organic) suggests that label attributes relating to the production of meat are influential in consumer's purchase intentions (Abrams et al., 2010); and as new labels such as 'raised without antibiotics' become available, perceptions of food choice are influenced in terms of the label's quality expectations (Karavolias et al., 2018). Consumer food choice is based upon the perception of quality however, perceived quality is not the same as genuine quality as the perceived quality is what the consumer believes the product is worth, often resulting in a "cost of ignorance" (Bowman et al., 2016). Nevertheless, many consumers do not want meat that is associated with antibiotics or drug residues and therefore, they want food labels to highlight the non-use of a production input or process (Umberger et al., 2009; Centner, 2016).

This mounting consumer pressure has led to the emergence of "antibiotic free" and associated labelling on meat products across several countries including the United States (US), Germany and Italy (Busch et al., 2020; Steede et al., 2020). In the US, a myriad of labelling such as "antibiotic free", "no antibiotics ever", and "no critically important antibiotics", are currently available on the market to specify that the animals have been reared without the use of antibiotics for non-medical reasons, i.e., growth promotion, and thereby indicate to consumers that the meat is safer (Driver, 2017a; Busch et al., 2020). However, in recent years similar labels have emerged on the UK market despite the introduction of the 2006 ban on antibiotics for growth promotion (RUMA, 2016). Furthermore, all livestock farming must abide by strict withdrawal periods following the treatment of an animal with antibiotics, to ensure that drug residues do not enter the food chain; subsequently, all food is antibiotic free (Farm Antibiotics, 2021). In addition, antimicrobial use is decreasing each year and antibiotic usage data is being successfully recorded via the electronic medicine book for pigs (eMB-Pigs), which is a Red Tractor scheme requirement (Driver, 2017b). Subsequently, the use of 'raised without antibiotics' labels can mislead and confuse consumers by unintentionally implying that the product is safer and that, by default, conventionally produced meat contains antibiotics and is therefore unsafe (Abrams et al., 2010; Driver, 2017a).

Of the few studies that have investigated consumer perceptions of 'raised without antibiotics' food labels, the majority have been conducted in the US (Abrams et al., 2010; Cervantes, 2015; Bowman et al., 2016; Centner, 2016; Ellison et al., 2017; Gulab, 2018; Karavolias et al., 2018; Steede et al., 2020). An abundance of studies focused on consumer preferences and perceptions towards more traditional characteristics, such as price, fat content, and appearance (Bernués et al., 2003) as well as a broader range of production-related attributes i.e., animal welfare standards and environmental friendliness (Verain et al., 2012; Denver et al., 2017; Grunert et al., 2018). In addition, empirical studies have attempted to estimate consumers' willingness to pay (WTP) for credence attribute food labels (Sanders et al., 2007; Yang & Renwick, 2019); particularly for products that have organic labelling and specific labels related to animal welfare (Hughner et al., 2007; Ellison et al., 2017). Previous research has also focused on consumer purchase intention, highlighting the relationship between sociodemographic characteristics such as age and gender, and willingness to buy products with credence attribute food labels (Zhang et al., 2012; Pelletier et al., 2013; Goddard et al., 2017).

Specific to antibiotic labelling, research has found that consumers place a premium on animal products produced without antibiotics (Lusk et al., 2006; Goddard et al., 2017; Busch et al., 2020); and exhibited a higher WTP for antibiotic free pork (Lusk et al., 2006; Goddard et al., 2017; Yang & Renwick, 2019) and milk (Olynk et al., 2010). Thus, while consumers are becoming more aware of label claims relating to food production methods, research shows that they have limited knowledge of what the labels mean, i.e., the food verification behind the label claim (Abrams et al., 2010). Antimicrobial use in livestock production supports good animal welfare by treating and controlling disease (Karavolias et al., 2018). However, according to a study conducted by Karavolias et al. (2018) assessing the impact of broilers raised without antibiotics, it was uncovered that consumers believe that purchasing poultry raised without antibiotics perpetuates good animal welfare. Consumers lack awareness towards the negative repercussions that banning or restricting antibiotics may have on animal welfare (Goddard et al., 2017); as 70% of US consumers purchase 'raised without antibiotics' labelled meat and poultry with the belief that it is healthier for the animal (Singer et al., 2019); and that it promotes good animal husbandry, while only 12% of consumers believe that it harms, or has no impact (9%) on animal welfare (Karavolias et al., 2018). As knowledge is an antecedent of beliefs (Howell et al., 2016) these results are indicative of an information gap in consumer understanding of animal welfare issues and knowledge surrounding agricultural antimicrobial use.

In order for consumers to make informed decisions when purchasing food, this knowledge gap should be addressed, particularly as ill-informed public opinion can have a negative influence on legislation and as such can be a detriment to the industry (Steede et al., 2020). Moreover, while antibiotic free livestock production may be an effective strategy to reduce AMR, eliminating antibiotic treatments is harmful for both animal production and welfare (Tang et al., 2017). Thus, 'raised without antibiotics' labels can have a negative influence on animal welfare if sick animals are denied antibiotic treatment in order to comply with the production methods behind this marketing label (Goddard et al., 2017). As a new addition to the UK market, it is unknown how consumers perceive 'raised without antibiotics' labelled products and whether the presence of such labels influence food choice. Research surrounding 'raised without antibiotics' labels in the UK has been exclusively based on analytical techniques, focusing on the identification of food-borne pathogens in 'raised without antibiotics' labelled foods (Tunsagool et al., 2021). Thus, to the best of our knowledge, this is the first study of its kind to investigate consumer knowledge and perceptions towards antimicrobial use in agriculture and willingness to buy 'raised without antibiotics' labelled meat. Understanding consumers' perceptions of credence attributes, such as the 'raised without antibiotics' label, can determine if they lack knowledge and understanding of the label and also what drives them to purchase 'raised without antibiotics' products (Yang & Renwick, 2019). In addition, it provides an insight into the values that consumers take into account i.e., environmental and ethical issues, when purchasing meat. Subsequently, this research will focus on UK consumers' perceptions towards meat raised without antibiotics, using the pictorial example of 'Spoilt Pig' bacon as it was the first meat bearing a 'raised without antibiotics' label to attain a place in the UK market.

Therefore, the objectives of the research were (1) to explore consumers' perceptions towards 'raised without antibiotics' labelled pork in comparison to traditional pork, (2) to investigate differences between sociodemographic factors: age, education, gender, and socio-economic status influence on consumers' perceptions, (3) to predict consumers' willingness to buy 'raised without antibiotics' labelled pork and the exploratory factors that influence purchase intention, and (4) to examine knowledge and awareness of antimicrobial use and AMR to identify if there is an information gap. The results offer insight into how the use of 'raised without antibiotics' food labels influence consumers' perceptions and attitudes of pork products, as well as how the label influences consumer purchasing decisions.

2. Methods

2.1. Data collection and participants

Cross-sectional consumer data were collected using a survey investigating various behavioural and psychological factors relating to

willingness to buy 'raised without antibiotics' labelled pork in a nationally representative sample of adults living in the UK. The sample (n = 1000) was quota-controlled in terms of age, sex, socio-economic status (SES), and region to match the known demographics of the population. Those working in the media, food safety, food processing or agriculture were excluded. In addition, those who had no shopping responsibility, purchased pork less than every few months and consumed pork less than every few months were also excluded. Data were collected by means of an online survey between 13th to 21st May 2020 by an online panel provider (Dynata™). Ethical approval was obtained from the School of Medicine, Dentistry and Biomedical Sciences Faculty Research Ethics Committee, Queen's University Belfast (Faculty REC Reference Number: MHLS 20_23). Respondents provided informed consent prior to completing the survey and all procedures were approved by the School of Biological Sciences and in accordance with the Declaration of Helsinki. An overview of participants sociodemographic characteristics is described in Table 1.

2.2. Questionnaire design

The questionnaire items were selected based upon previous research in consumer purchase intention as discussed. The questionnaire consisted of close-ended questions and was initially piloted among eight individuals to assess content and functionality (structure, filters, instructions, duration). At the outset, pork consumption and purchase frequency were measured as well as the importance of selected attributes in purchase decisions. Sociodemographic characteristics were then sought to ensure nationally representative quotas were attained. Following this, consumer knowledge of EU regulations, awareness of AMR, and antimicrobial use practices within both humans and animals were measured. Subsequently, participants were then presented with a pictorial example of a traditional pork product and a 'raised without antibiotics' labelled pork product, respectively (described in section 2.3) before completing items measuring behavioural beliefs, willingness to buy, and willingness to pay (WTP). Additionally, the degree of recognition was sought for each product and participants were shown product prices. Lastly, generalised trust and remaining sociodemographics (marital status, education, occupation status, household income, number of children and adults in household) were recorded.

2.3. Pictorial example of traditional and 'raised without antibiotics' labelled pork

Respondents were shown two examples of pork products currently available in supermarkets: a traditional pork product and a 'raised without antibiotics' labelled pork product. The pictorials were used to depict a product that consumers would be familiar with and also provide an example of a 'raised without antibiotics' labelled pork product which is now available on the UK market. Specifically, Spoilt Pig bacon was chosen as it was the first meat on the market with the 'raised without antibiotics' label and it was believed more consumers may have seen it. For comparison, the traditional product chosen was also a smoked bacon product. The pictorial examples highlighted the difference between each product due to the addition of the unique 'raised without antibiotics' label on the Spoilt Pig pork product. A visual aid showing traditional and 'raised without antibiotics' labelled pork were shown to respondents (Fig. 1) to illustrate the concept.

2.4. Measures

Items (listed in supplementary Table A) were scored on a 7-point Likert-type scale (1 = "strongly disagree", 7 = "strongly agree") unless otherwise indicated. All items were adapted from Spence et al. (2018)

Table 1
Sociodemographic details and characteristics of the study sample (n = 1000).

		Sample (%)
**Gender**	Male	49
	Female	51
**Age**	18–24 years	12
	25–34 years	17
	35–44 years	18
	45–54 years	18
	55–64 years	15
	65+ years	20
**Social Class**	ABC1[a]	51
	C2DE[b]	49
**Highest education level**	Primary education	1
	Secondary education (GCSE or equivalent)	21
	Secondary education (A-levels or equivalent)	16
	Vocational or technical qualifications (e.g. HND)	22
	University level	27
	Postgraduate level	11
	Doctorate, post-doctorate or equivalent	2
**Occupation**	Employed full-time (>30 h per week)	49
	Employed part-time ≤29 h per week)	14
	Full-time homemaker	5
	Unemployed	6
	Student	5
	Retired	21
**Marital status**	Married	51
	Single (never married)	27
	Widowed	3
	Divorced	5
	Separated	1
	Living with partner	13
**Household income**	Under £6999 per annum (less than £135 per week)	2
	£7000 - £14,999 per annum (£135 - £290 per week)	10
	£15,000 – £29,999 per annum (£290 - £580 per week)	27
	£30,000 – £59,999 per annum (£580 - £1150 per week)	36
	£60,000 + per annum (£1150 per week)	17
	Not sure	2
	Prefer not to say	6
**Household size**	1	18
	2	58
	3	13
	4	8
	5+	3
**Number of children under 16 in household**	0	72
	1	15
	2	10
	3+	3
**Frequency of pork purchase**	Daily	1
	Several times a week	14
	Several times a month	64
	Every few months	21
**Frequency of pork consumption**	Daily	1
	Several times a week	31
	Several times a month	54
	Every few months	14

[a] High social class: includes professional, managerial, technical, and skilled non-manual occupations.
[b] Low social class: includes skilled manual, partly skilled, and unskilled occupations.

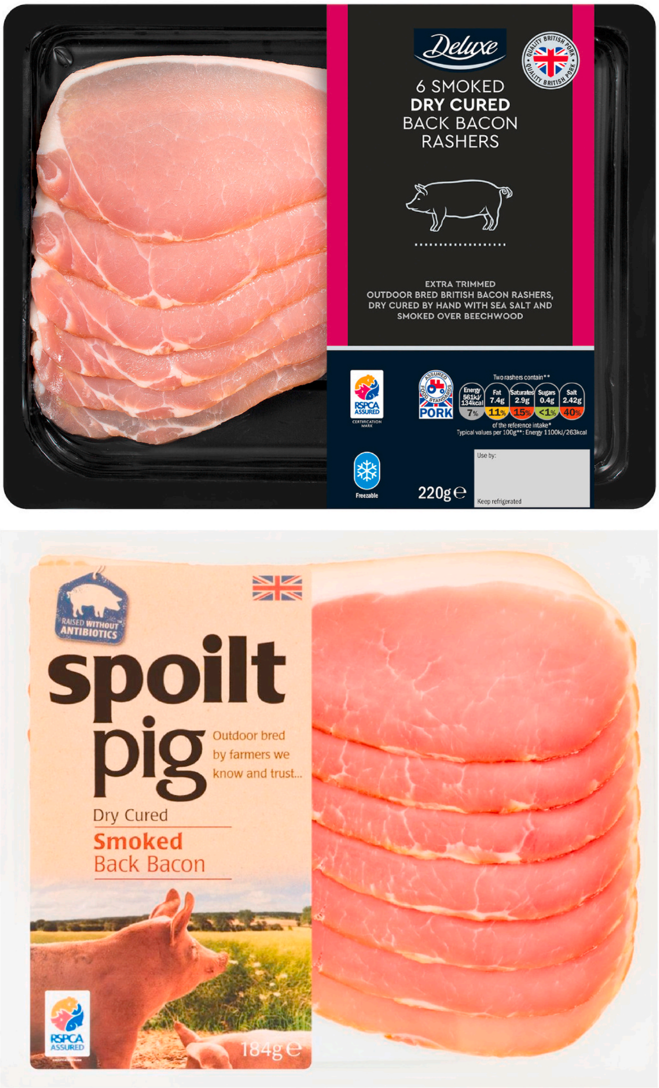

Fig. 1. Respondents were shown visual aids, a traditional pork product and a 'raised without antibiotics' labelled pork product, depicting the label illustrated on the 'raised without antibiotics' product.

unless otherwise indicated.

Pork purchasing habits: To measure the importance of pork attributes, thirteen items were used to identify and ascertain the factors most important to respondents when purchasing pork products (e.g., price, quality, quantity, appearance, origin, antibiotics used, organic, animal welfare practices, place of purchase, the brand, healthiness, environmental friendliness, type of packaging).

Knowledge of EU regulations: Respondent's knowledge of EU regulated pork was evaluated by five items (constructed by the author) and measured using a dichotomous scale (true/false).

Behavioural beliefs towards traditional pork: To measure behavioural beliefs towards a traditional pork product currently available in supermarkets, eight statements were measured (e.g. traditional pork is: healthy, expensive, tasty, easy to find, of good quality, safe to eat, produced to ensure that animal rights have been respected, a product that does not contain antibiotics).

Behavioural beliefs towards 'raised without antibiotics' labelled pork: To measure behavioural beliefs towards 'raised without antibiotics' labelled pork, respondents were presented with the same eight statements that compared 'raised without antibiotics' labelled pork to traditional pork (e.g., 'raised without antibiotics' labelled pork will likely be: healthier, more expensive, tastier, easier to find, of more satisfying quality, safer to eat, produced with higher animal welfare standards, free from antibiotics).

Willingness to buy: To measure willingness to purchase 'raised without antibiotics' labelled pork, participants responded to the statement: "I would be more willing to buy products labelled 'raised without antibiotics' than those without this label."

WTP: Respondents indicated if they would be willing to pay more for 'raised without antibiotics' labelled pork directly through the question: "I would be willing to pay more for this product than a traditional product without the 'raised without antibiotics' label."

Awareness of AMR: Awareness towards AMR was assessed by two dichotomous scale (yes/no) items (adapted from Goddard et al., 2017): "have you heard of antibiotic or antimicrobial resistance?" and "do you know what antimicrobial resistance is?"

Perceptions of antimicrobial use: Perceptions towards human and animal antimicrobial use practices were assessed by eleven items (adapted from Goddard et al., 2017); three relating to human practices and eight in relation to animal practices.

Generalised trust: Respondents rated the extent to which they trust others with four items (unpublished): "most people are basically honest", "most people are trustworthy", "most people are basically good and kind" and "most people are trustful of others".

2.5. Data analysis

All data were analysed using IBM SPSS Statistics version 26.0 (IBM Corporation, Armonk, NY, USA), with a p-value $p \leq 0.05$ considered to be significant.

Construction of variable scales: Each of the 44 Likert-type questionnaire items (see supplementary Table A) were entered into a maximum likelihood factor analysis with Direct Oblimin rotation, supressing factor loadings < 0.3, to identify a measurement model. The item loadings were then examined to ensure that a clean solution was attained and Cronbach's α coefficient was calculated to investigate the internal reliability of each construct (Table 2), with an α value of > 0.70 deemed acceptable (Kline, 1999). The items within each construct were then averaged by computing a mean of the loading items and scales were constructed. Scores of each scale ranged from a minimum of 1 to a maximum of 7, with higher values signifying stronger levels of the construct. Finally, resulting scales were labelled based upon their content and Pearson correlations were computed to measure the strength of the relationship between constructs within the model.

Descriptive analysis: Descriptive statistics (mean and SD) were used to explore the data (see supplementary Table A). Independent samples t-tests were used to investigate the differences between the sociodemographic characteristics of participants (age, gender, education, and SES) and the various model constructs (Table 3).

Regression analysis: Hierarchical multiple regression analyses were used to examine the association between predictor variables and willingness to purchase 'raised without antibiotics' labelled pork. In step 1, exploratory predictor variables were entered while sociodemographic characteristics were entered in step 2. In each regression, the bootstrap method was applied to account for non-normality of residuals (based upon P–P plot). The bootstrap method has been previously applied in past consumer studies.

2.5.1. Factor analysis and construction of variable scales

Generalised trust was the only variable that yielded a unifactorial solution ($\alpha = 0.93$). Pork purchasing habits yielded a three-factor solution identified as *Animal welfare qualities* ($\alpha = 0.88$), *Extrinsic qualities* ($\alpha = 0.75$), and *Marketing qualities* ($p = 0.58$). Traditional behavioural beliefs yielded a two-factor solution however, the item 'expense' had a low factor loading (< 0.4) and was therefore used to create a separate factor. Subsequently, traditional beliefs comprised three factors: *Product quality* ($\alpha = 0.86$), *Animal welfare* ($\alpha = 0.73$), and *Expense*. Similarly, 'raised without antibiotics' behavioural beliefs also yielded a two-factor

Table 2
Factor analysis subscale reliability– standardised factor loading and Cronbach's alpha.

	Factors	Codes	Alpha	Factor loadings	Mean (SD)
**Pork purchasing habits**	Animal welfare qualities	hab 1	0.88	0.51	5.00 (1.13)
		hab 2		0.66	
		hab 3		0.69	
		hab 4		0.90	
		hab 5		0.49	
		hab 6		0.87	
		hab 7		0.54	
	Extrinsic qualities	hab 8	0.75	0.52	5.55 (0.97)
		hab 9		0.73	
		hab 10		0.67	
		hab 11		0.71	
	Marketing qualities	hab 12	0.58*	0.51	4.77 (1.25)
		hab 13		0.91	
**Traditional beliefs**	Product quality	bel 3	0.86	0.83	5.37 (0.88)
		bel 4		0.64	
		bel 5		0.90	
		bel 6		0.75	
	Animal welfare	bel 1	0.73	0.40	4.93 (1.13)
		bel 7		0.82	
		bel 8		0.76	
	Expense	bel 2	–	0.14	4.63 (1.26)
**'Raised without antibiotics' pork beliefs**	Product quality	rwa 3	0.79	0.71	4.80 (1.06)
		rwa 4		−0.65	
		rwa 5		−0.66	
		rwa 6		0.67	
	Animal welfare	rwa 1	0.78	0.56	5.59 (1.12)
		rwa 7		0.71	
		rwa 8		0.73	
	Expense	rwa 2	–	0.54	5.64 (1.21)
**Perceptions of antimicrobial use**	Personal concern	per 1	0.65	0.71	5.05 (1.19)
		per 2		0.67	
		per 3		0.50	
	Animal welfare standards	per 4	0.75*	0.85	5.67 (1.23)
		per 5		0.88	
	Animal usage acceptance	per 6	0.71	0.68	4.25 (1.13)
		per 8		0.70	
		per 9		0.64	
	Animal concern	per 7	0.55	0.50	4.12 (1.09)
		per 10		0.61	
		per 11		0.55	
**Generalised trust**	Unifactorial	gen 1	0.93	0.92	4.58 (1.17)
		gen 2		0.94	
		gen 3		0.86	
		gen 4		0.78	

*Inter-item correlation ($p < 0.01$).

solution however, for practicality and to enable comparisons between groups, items were organised according to the traditional belief structure solution. Thus, group one consisted of four items relating to the quality of pork raised without antibiotics (and was therefore labelled *Product quality*, α = 0.79), group two consisted of three attributes relating to animal welfare practices (and was therefore labelled *Animal welfare*, α = 0.78) and group three similarly omitted expense of 'raised without antibiotics' labelled pork to create a separate factor (labelled *Expense*). Perceptions towards antimicrobial use practices contained four factors, identified as *Personal concern* (α = 0.65), *Animal welfare standards* (p = 0.75), *Animal antimicrobial use acceptance* (α = 0.71), and *Animal concern* (α = 0.55). Due to unacceptable internal reliability, the factor Animal concern was removed from the analysis. Although the internal reliability of Personal concern was also < 0.7 (α = 0.65), it was deemed acceptable for analysis as recommended by Ursachi et al. (2015) as the a is within the range of 0.6 – 0.7. All factor items and internal reliability values are presented in Table 2.

3. Results

3.1. Description of demographic information

In total, 1000 participants aged 18–92 years old (M = 46.8, SD = 16.8) completed the survey (see Table 1 for sociodemographic details). Fifty one percent of participants were female, 50% were in full time employment, and 27.5% were educated to university level. All respondents shared at least some shopping responsibility. Two out of three participants stated that they purchase pork occasionally (1–4 times a month) and 54% reported occasional pork consumption (1–4 times a month). While 79% of participants were familiar with the traditional pork product, only 22% were familiar with the 'raised without antibiotics' labelled product. Regarding personal antimicrobial use, 91% have taken antibiotics in their lifetime and of these respondents, 30.5% have taken antibiotics in the past 12 months. In addition, 16% have experienced a time when antibiotics haven't worked.

3.2. Consumer perceptions and willingness to buy 'raised without antibiotics' labelled pork

When purchasing pork, participants considered extrinsic qualities (M = 5.55, see Table 2) (i.e., price, quality, quantity, and appearance) as the most important attributes influencing their purchase intention, followed by qualities concerning animal welfare (M = 5.00). Marketing qualities (M = 4.77) relating to the place of purchase and the brand were considered the least important attributes however, a neutral to positive score was similarly recorded. There was a significant difference among male and female consumers regarding animal welfare qualities (see Table 3), as female respondents (M = 5.10) were significantly more concerned about animal welfare standards when purchasing pork compared to their male (M = 4.89) counterparts ($p = 0.002$).

Further, participants recorded a strong belief that traditional pork is of good quality (item scored above 5); with product price and animal welfare standards involved in production beliefs to be neutral (M = 4.63 and 4.81, respectively). There were significant differences in behavioural beliefs towards traditional pork based on consumer education and age. Those with education to vocational level believed the product was not only good quality ($p = 0.018$) but that traditional pork is produced to ensure that animal rights have been respected ($p = 0.041$). Significant differences were also noted between age groups and beliefs as older participants had more positive beliefs towards traditional product quality ($p = 0.001$), while younger participants believed this product to be expensive ($p = 0.045$).

In contrast, participants strongly believed that 'raised without antibiotics' labelled pork would be more expensive than traditional pork and that it would also be produced with higher animal welfare standards (items scored above 5). Additionally, consumers shared the belief that 'raised without antibiotics' labelled pork would be of lesser quality (M = 4.90) than traditional pork (M = 5.55). Further, participants believe that 'raised without antibiotics' labelled pork will not be easy to find in supermarkets (M = 3.77). There were significant differences between behavioural beliefs based on gender as females believed 'raised without antibiotics' labelled pork to be more expensive, of better quality and also to be produced with higher animal welfare standards in comparison to traditional pork than men (all items $p < 0.01$).

Participants' willingness to buy 'raised without antibiotics' labelled pork was high (M = 5.09); however, consumers' WTP a premium price for the product was lower (M = 4.65). When presented with the price of 'raised without antibiotics' labelled pork currently retailing at £2.75/184g (£1.49/100g); 68% of respondents stated that they would most likely purchase the traditional product, retailing at £1.89/220g (85.9p/

Table 3
Differences in model constructs by sociodemographic subgroups.

	Age Mean (SD)		Gender Mean (SD)		SES Mean (SD)		Education Mean (SD)	
**Variables**	< 45	46 +	Male	Female	ABC1	C2DE	≤ Secondary[a]	≥ Tertiary[b]
Pork purchasing habits								
Animal welfare qualities	5.02 (1.13)	5.00 (1.13)	4.89 (1.18)	**5.10 (1.07)****	5.03 (1.14)	4.96 (1.12)	4.95 (1.17)	5.03 (1.11)
Extrinsic qualities	5.55 (0.97)	5.56 (0.97)	5.51 (0.98)	5.60 (0.96)	5.53 (0.95)	5.58 (0.99)	5.62 (1.02)	5.52 (0.94)
Marketing qualities	4.79 (1.29)	4.75 (1.21)	4.70 (1.28)	4.84 (1.21)	4.79 (1.25)	4.76 (1.25)	4.83 (1.27)	4.74 (1.24)
Knowledge of EU regulations[c]	3.15 (1.02)	**3.42 (0.98)****	3.37 (1.00)	**3.21 (1.01)***	3.27 (1.02)	3.31 (1.00)	3.26 (1.02)	3.31 (1.00)
Beliefs (traditional)								
Product quality	5.27 (0.86)	**5.46 (0.89)****	5.42 (0.90)	5.32 (0.86)	5.39 (0.87)	5.34 (0.89)	5.45 (0.90)	**5.32 (0.86)***
Animal welfare	4.88 (1.18)	4.99 (1.08)	4.90 (1.13)	4.97 (1.13)	4.94 (1.09)	4.93 (1.17)	5.03 (1.11)	**4.88 (1.14)***
Expense	4.71 (1.28)	**4.55 (1.24)***	4.61 (1.27)	4.64 (1.25)	4.68 (1.26)	4.57 (1.26)	4.60 (1.26)	4.64 (1.26)
Have you seen?[d]	1.76 (0.43)	**1.82 (0.38)***	1.81 (0.40)	1.78 (0.41)	1.80 (0.40)	1.79 (0.41)	1.82 (0.39)	1.78 (0.42)
Would you buy?[d]	1.89 (0.32)	1.90 (0.30)	1.91 (0.28)	1.88 (0.33)	1.89 (0.31)	1.90 (0.30)	1.91 (0.28)	1.88 (0.32)
Beliefs ('raised without antibiotics')								
Product quality	4.82 (1.05)	4.78 (1.08)	4.69 (1.09)	**4.90 (1.03)****	4.84 (1.07)	4.75 (1.06)	4.82 (1.10)	4.78 (1.04)
Animal welfare	5.60 (1.09)	5.59 (1.15)	5.48 (1.16)	**5.70 (1.08)****	5.65 (1.09)	5.53 (1.15)	5.60 (1.14)	5.59 (1.11)
Expense	5.65 (1.20)	5.64 (1.21)	5.53 (1.23)	**5.75 (1.17)****	5.71 (1.18)	5.58 (1.23)	5.58 (1.34)	5.69 (1.11)
Willingness to buy	5.11 (1.33)	5.07 (1.42)	4.93 (1.42)	**5.23 (1.32)****	5.14 (1.38)	5.03 (1.38)	5.08 (1.39)	5.09 (1.37)
WTP	4.73 (1.50)	4.57 (1.61)	4.44 (1.63)	**4.84 (1.47)****	4.73 (1.59)	4.56 (1.53)	4.63 (1.57)	4.66 (1.56)
Awareness of AMR[e]	0.96 (0.91)	**0.84 (0.89)***	0.86 (0.90)	0.94 (0.90)	1.02 (0.91)	**0.78 (0.88)****	0.71 (0.85)	**1.02 (0.91)****
Perceptions of antimicrobial use								
Personal concern	5.07 (1.13)	5.03 (1.25)	4.93 (1.26)	**5.16 (1.11)****	5.14 (1.19)	**4.95 (1.18)***	4.93 (1.23)	**5.12 (1.17)***
Animal usage acceptance	4.43 (1.13)	**4.09 (1.10)****	4.28 (1.11)	4.23 (1.15)	4.36 (1.16)	**4.14 (1.08)****	4.24 (1.10)	4.26 (1.14)
Animal welfare standards	5.55 (1.21)	**5.81 (1.23)****	5.60 (1.27)	**5.77 (1.18)***	5.68 (1.22)	5.70 (1.24)	5.71 (1.23)	5.68 (1.23)

n = 1000.
All items were scored on a 7-point Likert-scale unless otherwise indicated.
Significantly different ($p < 0.05$*; < 0.01**; bold numbers highlight significance).
[a] ≤ Secondary: includes primary education, secondary education (GCSE/O-Level/CSE or equivalent), secondary education (A-Level or equivalent), and vocational or technical qualifications completed (e.g. HND, NVQ).
[b] ≥ Tertiary: includes university education completed (first degree e.g. BA, BSc), postgraduate education completed (e.g. Masters), and doctorate, post-doctorate or equivalent (Higher degree).
[c] Scale 0-5 based on 5 true/false questions.
[d] Scale 1-2 based on yes/no questions where 1 = no and 2 = yes.
[e] Scale 0-2 based on yes/no questions where 0 = low awareness and 2 = high awareness.

100g). In relation to the 'raised without antibiotics' labelled pork product, female respondents had a significantly greater willingness to buy and also pay more for 'raised without antibiotics' labelled pork, compared to their male counterparts ($p = 0.001$).

Participants had a high level of knowledge of EU regulations (i.e., mean = 3.29 out of a possible 5, SD = 1.01). However, awareness of AMR was limited as while the majority had heard of AMR (52%), only 38% of respondents knew what AMR is. There were significant differences in both knowledge of EU regulations and awareness of AMR with age; with older respondents having a higher level of knowledge of EU regulations ($p < 0.001$) and, in contrast, younger respondents having significantly more awareness of AMR ($p = 0.038$).

Participants scored high on perceptions of antimicrobial use practices, showing a high level of personal concern towards AMR and a high consideration for animal welfare standards (all items score above 5). Participants thought that too many antibiotics from the doctor can cause AMR and that AMR will interfere with disease treatment. In addition, adherence to animal welfare standards and ensuring animals do not experience pain were considered important attributes. While reporting a more moderate score (M = 4.25) participants were still positively accepting of antimicrobial use in livestock production. In comparison, negative perceptions towards domestic pets acting as a potential source of AMR transfer (M = 3.45) indicate that respondents do not consider domestic animals to be a reservoir or spread resistant bacteria.

There were significant differences in perceptions of antimicrobial use practices between various demographic characteristics. Respondents from the lower SES group ($p = 0.016$) were more concerned with personal risk associated with antimicrobial use. In addition, those educated beyond university level were also more concerned than those with lower levels of education ($p = 0.018$). The lower SES group were also more accepting of antimicrobial use in livestock production ($p = 0.002$). There were significant differences in perceptions based on gender where female respondents were more concerned about personal antimicrobial use risks ($p = 0.002$) and showed greater consideration for high animal welfare standards ($p = 0.026$). Contrastingly, older respondents were significantly more concerned with high levels of animal welfare ($p = 0.001$), while respondents from the younger age group were more accepting of antimicrobial use during animal production ($p < 0.001$).

3.3. Predicting willingness to buy 'raised without antibiotics' labelled pork

Correlations between exploratory constructs (as shown in Table 4) show that all constructs except knowledge of EU regulations, awareness of AMR, beliefs concerning price of traditional pork, age, education, and SES of participants, were significantly correlated with willingness to purchase 'raised without antibiotics' labelled pork. In relation to behavioural beliefs, pork quality had the strongest relationship with willingness to buy, indicating that those who perceived the 'raised without antibiotics' labelled product to be of better quality than traditional pork, were more likely to purchase it. Moderate positive correlations were also observed between willingness to buy and the following constructs: pork purchasing habits based on animal welfare qualities, 'raised without antibiotics' labelled pork beliefs concerning animal

Table 4
Correlations between willingness to buy and exploratory constructs contained within the models.

Exploratory constructs	1	2	3	4	5	6	7	8	9	10	11	12	13	14	15	16	17	18	19	20
1. Willingness to buy	–																			
2. Pork purchasing habits (animal welfare qualities)	0.43**	–																		
3. Pork purchasing habits (extrinsic qualities)	0.08*	0.32**	–																	
4. Pork purchasing habits (marketing qualities)	0.27**	0.60**	0.24**	–																
5. Knowledge of EU regulations	−0.05	−0.06*	0.03	−0.07*	–															
6. Traditional pork beliefs (product quality)	0.31**	0.13**	0.21**	0.09**	0.13**	–														
7. Traditional pork beliefs (animal welfare)	0.33**	0.27**	0.12**	0.29**	0.17**	0.59**	–													
8. Traditional pork beliefs (expense)	0.06	0.01	0.11**	0.04	−0.09**	0.13**	0.20**	–												
9. RWA[a] pork beliefs (product quality)	0.68**	0.44**	0.08*	0.36**	−0.06*	0.34**	0.47**	0.13**	–											
10. RWA[a] pork beliefs (animal welfare)	0.67**	0.35**	0.18**	0.21**	0.02	0.43**	0.37**	0.10**	0.69**	–										
11. RWA[a] pork beliefs (expense)	0.15**	0.04	0.16**	−0.04	−0.006	0.27**	0.09**	0.20**	0.23**	0.41**	–									
12. Awareness of AMR	0.01	0.03	−0.02	−0.10**	0.002	0.04	−0.09**	−0.03	−0.07*	0.06	0.10**	–								
13. Perceptions of AMU[b] (personal concern)	0.34**	0.28**	0.05	0.11**	−0.02	0.22**	0.20**	0.13**	0.30**	0.35**	0.23**	0.29**	–							
14. Perceptions of AMU[b] (animal usage acceptance)	−0.10**	−0.08*	0.004	0.06	0.02	0.18**	0.22**	0.16**	0.06*	0.008	0.06*	−0.05	0.009	–						
15. Perceptions of AMU[b] (animal welfare standards)	0.43**	0.46**	0.16**	0.23**	0.02	0.36**	0.34**	0.04	0.38**	0.47**	0.22**	0.10**	0.40**	0.005	–					
16. General trust	0.20**	0.25**	0.06	0.23**	0.09**	0.23**	0.33**	0.07*	0.26**	0.20**	0.02	0.005	0.16**	0.18**	0.25**	–				
17. Age	−0.01	−0.02	0.003	−0.02	0.13**	0.07*	0.10**	−0.06*	−0.05	0.03	−0.002	−0.07*	−0.02	−0.15**	0.11**	0.13**	–			
18. Education	0.003	0.04	−0.05	−0.04	0.02	−0.06	−0.09**	0.02	−0.02	−0.01	0.04	0.17**	0.08*	0.007	−0.01	0.01	−0.14**	–		
19. Gender	0.11**	0.10**	0.05	0.06	−0.08*	−0.03	−0.02	0.01	0.09**	0.11**	0.09**	0.05	0.10**	−0.02	0.07*	−0.04	−0.23**	−0.005	–	
20. Socio-economic status	−0.04	−0.03	0.02	−0.01	0.02	−0.05	0.01	−0.05	−0.05	−0.04	−0.05	−0.13**	−0.08*	−0.10**	0.006	−0.01	0.25**	−0.24**	−0.02	–

*$p < 0.05$; **< 0.01; bold numbers highlight significance.
[a] RWA = Raised without antibiotics.
[b] AMU = Antimicrobial use.

welfare, perceptions towards personal risk of antimicrobial use, perceptions towards animal welfare standards, and beliefs concerning product quality and animal welfare of traditional pork.

Together exploratory variables account for 58% (based on R^2_{adj}) of the variance in willingness to buy 'raised without antibiotics' labelled pork (Table 5). Beliefs on animal welfare and product quality relating to 'raised without antibiotics' labelled pork were the main predictors of willingness to buy, followed by pork purchasing habits based on animal welfare qualities, and perceptions of antimicrobial use relating to personal concerns and animal welfare standards. Therefore, having more favourable beliefs towards animal welfare (ß = 0.47) and product quality (ß = 0.46) associated with 'raised without antibiotics' labelled pork, priority of animal welfare when purchasing pork (ß = 0.14), being personally concerned about antimicrobial use (ß = 0.09) and having favourable perceptions of animal welfare standards during production (ß = 0.06), were associated with a greater willingness to purchase 'raised without antibiotics' labelled pork. Further, acceptance of animal antimicrobial use (ß = −0.14), price (ß = −0.13), and pork purchasing habits focused on extrinsic qualities (ß = −0.07) had a negative influence on willingness to buy 'raised without antibiotics' labelled pork. Therefore, consumers with favourable perceptions towards antimicrobial use in animals, who believe 'raised without antibiotics' labelled pork is expensive and consider extrinsic qualities as important attributes when purchasing pork, were less likely to purchase 'raised without antibiotics' labelled pork.

When the model was extended with the addition of age, education, gender, and SES, hierarchical multiple regression revealed that the variance explained (based on R^2_{adj}) in willingness to purchase 'raised without antibiotics' labelled pork remained constant at 58% ($p < 0.001$). Thus, sociodemographic characteristics did not explain any additional variance in the prediction of willingness to purchase. In the final model, all variables remained significant drivers of willingness to buy 'raised without antibiotics' labelled pork (see Fig. 2 for final model).

Table 5
Bootstrapped unstandardised regression coefficients (ß) for both model 1 exploratory constructs and model 2 extended sociodemographic constructs from regression analysis predicting consumers' willingness to buy 'raised without antibiotics' labelled pork.

Exploratory constructs	Model 1	Model 2
Purchasing habits (animal welfare qualities)[a]	**0.14*****	**0.13****
Purchasing habits (extrinsic qualities)[a]	**−0.07***	**−0.07***
Purchasing habits (marketing qualities)[a]	−0.02	−0.02
Knowledge of EU regulations[b]	−0.04	0.04
Traditional pork beliefs (product quality)[a]	0.09	0.09
Traditional pork beliefs (animal welfare)[a]	−0.03	−0.02
Traditional pork beliefs (expense)[a]	0.002	0.001
'Raised without antibiotics' pork beliefs (product quality)[a]	**0.46*****	**0.45*****
'Raised without antibiotics' pork beliefs (animal welfare)[a]	**0.47*****	**0.47*****
'Raised without antibiotics' pork beliefs (expense)[a]	**−0.13*****	**−0.13*****
Awareness of AMR[c]	−0.03	−0.03
Perceptions of antimicrobial use (personal concern)[a]	**0.09****	**0.09****
Perceptions of antimicrobial use (animal usage acceptance)[a]	**−0.14*****	**−0.15*****
Perceptions of antimicrobial use (animal welfare standards)[a]	**0.06***	**0.07***
Generalised trust[a]	0.00	0.004
Age		−0.06
Education		0.03
Gender		0.06
Socio-economic status		−0.01
R^2_{adj}	0.58	0.58
Model F	**92.90*****	**73.40*****
ΔR^2	–	0.001
Df	984	980

*$p \leq 0.05$; **$p < 0.01$; ***$p < 0.001$; bold text highlights significance.
[a] Mean of variable items measured on a 7-point Likert scale; higher scores indicative of stronger (i.e., more positive) levels of the construct.
[b] Knowledge scale 0–5 based on 5 true/false questions. 0 = low knowledge and 5 = high knowledge.
[c] Awareness of AMR scale 0–1 based on yes/no questions where 0 = low awareness and 1 = high awareness.

4. Discussion

Food labels enable producers to deliver product information regarding credence quality attributes (i.e., antibiotic free) to the consumer. The objectives of this survey study were to explore consumer perceptions and willingness to buy 'raised without antibiotics' labelled pork in the UK, and to identify predictors of purchase intention of 'raised without antibiotics' labelled pork.

4.1. Consumers' perceptions towards 'raised without antibiotics' labelled pork in comparison to traditional pork

The present study suggests that consumers generally have positive beliefs towards traditional pork, particularly relating to product quality and the animal welfare standards involved during production. Contrastingly, participants shared a belief that 'raised without antibiotics' labelled pork would be more expensive than traditional pork and produced with higher animal welfare standards however, they believed it would be of average quality, less than that of traditionally produced pork. In addition, participants had the belief that 'raised without antibiotics' labelled pork would not be easy to locate in supermarkets. This insight into consumer beliefs surrounding 'raised without antibiotics' labelled pork suggests that more transparency is needed as the label does not refer to increased animal welfare standards, yet consumers mistakenly identify livestock production free from antibiotics as one that has beneficial impact on animal welfare. Similarly, in an organic marketing report by Schroeder et al. (2014) it was found that consumers also mistakenly associate the USDA organic seal as 'healthier', 'safer', and 'more nutritious' despite the label having no relation to food safety. Yet, it must also be noted that price is an integral purchase inhibiting factor for many consumers; in a study conducted by Abrams et al. (2010) investigating consumer perceptions towards organic and all-natural pork products, it was reported that consumers had similar positive associations towards these labelled products, with exceptions relating to price. Therefore, as 'raised without antibiotics' labelled pork is considered to be more expensive than traditional pork, this could discourage consumers from purchasing it.

Our results also revealed that gender was significant, meaning that women believed 'raised without antibiotics' labelled pork is of better quality, produced with higher animal welfare standards, and is also more expensive than traditional pork. As women are more likely to be the primary grocery shoppers in a household (Zingg & Siegrist, 2012); this may suggest that they have a greater awareness of the products currently available in supermarkets and the pricing associated with product labels.

While various studies have indicated that consumer's awareness influences food choice (Yiridoe et al., 2005; Gotschi et al., 2009; Abrams et al., 2010; Rahnama et al., 2017); consumer perceptions are considered one of the most important factors in terms of purchasing decisions, often driving consumer intention to buy (Wheatley, 2001). In the present study, participants perceptions varied depending on antimicrobial use practices. Significant differences were observed in risk perception as low SES participants and those with high levels of education were more concerned with personal risks associated with antimicrobial use. This finding is somewhat contradictory however, a possible explanation for this encompasses a finding outlined by Inglis et al. (2005) who determined that women of low SES tended to report certain eating patterns that were less healthy. Thus, as individuals of a low SES are aware of their unhealthy eating patterns, they may have more awareness of the associated risks and subsequently, hold higher levels of personal

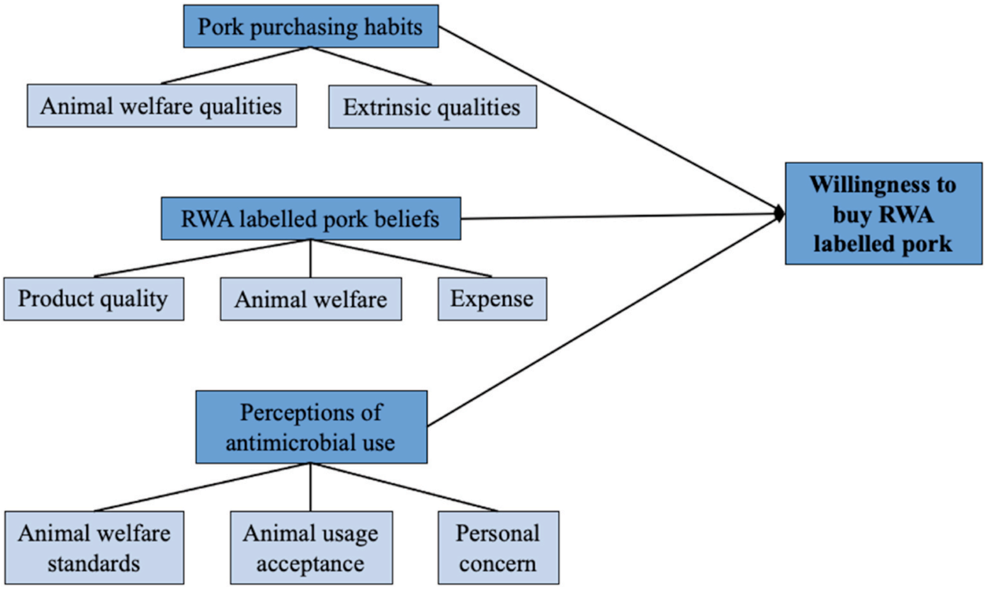

Fig. 2. Final regression model showing the exploratory factors influencing willingness to buy 'raised without antibiotics' (RWA) labelled pork.

concern. Generally, personal risk perception towards AMR and consideration towards animal welfare standards was high. Participants personal risks encompassed perceptions surrounding medical antimicrobial use and opinions that too many antibiotics from the doctor can cause AMR and thus, that they would be unable to treat illness. This finding is consistent with other studies which demonstrate that there is a high degree of concern among people about being affected by AMR in the future (Goddard et al., 2017); with overprescribing of antibiotics by medical professionals being identified as the major cause of AMR development (Steede, 2020). In comparison to a study conducted by Goddard et al. (2017) among German and Canadian participants, a slightly lesser percentage of our sample experienced a time when an antibiotic treatment has failed, and they have been told so by a medical professional (16% versus 20%). Although this is a smaller percentage of the sample population, it is still a large proportion of the UK population who have been affected by AMR.

In addition to personal concerns, participants also exhibited high levels of consideration towards animal welfare standards during livestock production, perceiving the absence of animal pain as an important factor influencing their willingness to buy. However, previous research surrounding consumer interest in animal welfare aspects of livestock production has shown that consumer perceptions vary significantly. In a study conducted by Grunert et al. (2018) it was uncovered that German respondents perceive animal welfare relating to free mobility for the sow as the most important attribute relating to production, however, Polish respondents believe that animal welfare attributes are relatively unimportant. Overall, the results of this study determined that the most desired credence quality attributes were those providing individual benefit rather than societal or animal benefits (Grunert et al., 2018). Busch et al. (2020) also found that while US respondents had positive perceptions towards antimicrobial use for animal welfare purposes, the German sample did not acknowledge such advantages. Although our study shows that consumers do value animal welfare standards during pork production, research in this area varies substantially and thus, more research should be conducted; particularly as it can be more difficult to justify the use of antibiotic treatments in the agricultural sector if consumers do not value animal welfare.

In contrast to consumers' perceptions surrounding personal antimicrobial use and animal welfare, participants had more neutral perceptions towards antimicrobial use in livestock production. In line with findings reported by Busch et al. (2020), participants from Germany, Italy, and the US also perceived greater risks from antimicrobial use in livestock production; however, it was considered that consumers perceive the use of veterinary drugs as something out of their control and therefore, more harmful compared to procedures that are within their control. Subsequently, consumers may perceive the agricultural sector to use antibiotics irresponsibly and as a result, form negative views towards the use of antibiotics in livestock production. These neutral views uncovered towards animal antimicrobial use contradict the high importance participants place on animal welfare standards as antimicrobial use would be an integral practice in maintaining these standards. Indeed, Karavolias et al. (2018) found that prohibiting access to antibiotics in broiler production led to increased risk and severity of specific diseases as broilers raised without antibiotics had a greater incidence of eye burns, foot lesions, and airsacculitis occurring, in comparison to those given antibiotics. Additionally, Gaucher et al. (2015) discovered that antibiotic free production is associated with adverse effects on performance and gut health, indicative of the negative effects on overall animal welfare. Thus, the production of pork raised without antibiotics may be detrimental to animal health and welfare outcomes.

4.2. *Knowledge and awareness of antimicrobial use and resistance*

In general, consumers have high levels of knowledge towards EU regulations however, their awareness of AMR was limited as half of the sample population had heard of it and only 38% knew what AMR is. Additionally, our research findings are in line with previous research which suggests that consumers may be unaware of the vital role that antibiotics have in the livestock production sector and thus, lack knowledge towards current agricultural antimicrobial use practices. The present study shows that while participants are concerned about the negative effects of antimicrobial use in livestock production, they perceive this risk to occur entirely from consumption of food producing animals treated with antibiotics, as they did not perceive domestic pets to be a potential source of AMR transfer. Further demonstrating a gap in consumer knowledge surrounding the transfer of resistant bacteria between animals and humans. These results compare favourably with other studies conducted by Abrams et al. (2010), Bowman et al. (2016) and Jaja (2017) which have similarly shown that there are gaps in consumers' knowledge. According to Abrams et al. (2010), consumers do not understand why or how additives in meat are bad for them, however, they are more inclined to buy food products with the "no" labelling premise. This suggests that consumers have limited

understanding of food threats but rather rely on product packaging for marketing claims. Subsequently, Bowman et al. (2016) raised the question, "what if consumers do not have well-defined preferences about antibiotic use on farms, or do not understand the issue or information provided to them via a label claim?" Thus, an uneducated public could drive unethical production and cause damage to our food industry, reinforcing consumer mistrust in the industry. The present study shows that animal welfare is important to consumers and therefore, there is a need to communicate the role of antibiotics in livestock production with a specific focus on welfare, highlighting that the production of meat without antibiotics can be detrimental and provides no further benefit to human health than conventionally produced pork.

4.3. Consumers' willingness to buy 'raised without antibiotics' labelled pork

Livestock production free from antibiotics is less efficient (i.e., average daily gain, feed conversion ratio, mortality, and yield) than conventional production as the cost of producing a kg or lb of meat is higher and therefore, consumers have to pay more for meat raised without antibiotics (Cervantes, 2015). Previous studies have found that consumers place substantial premiums on pork produced without antibiotics (Lusk et al., 2006); and are WTP extra to guarantee that the meat they consume is free from antibiotics (Grannis & Thilmany, 2001). Wheatley (2001) uncovered that on average consumers were WTP an additional 10%–37% for organic produce, and as such, we anticipated that consumers would be WTP a premium for 'raised without antibiotics' labelled pork. Interestingly, while the majority of participants stated that they would buy 'raised without antibiotics' labelled pork, they had more neutral views towards paying a premium for the product. This finding is consistent with that of Goddard et al. (2017) whereby 9% of German participants and 15% of Canadian participants were also unwilling to pay extra for products from animals treated with 60% less antibiotics. Thus, overall, participants stated that they would most likely purchase traditional pork based solely on the price of 'raised without antibiotics' labelled pork being £0.64 more expensive per 100g.

Despite this, it was found that female participants had a significantly greater willingness to buy and pay a premium for 'raised without antibiotics' labelled pork. This result may relate to our previous finding that women believe 'raised without antibiotics' labelled pork is better quality and produced with increased animal welfare standards, if these attributes are considered to be of value to the consumer, it will positively influence their purchase intention. Zingg and Siegrist (2012) yielded similar results, ultimately reflecting differences in risk perception between male and female consumers. Women tend to express greater concern for food safety (Zingg & Siegrist, 2012) and healthy eating (Wardle et al., 2004); and they take information about health and nutrition more seriously than their male counterpart (Verbeke and Lui, 2014).

4.4. Factors influencing willingness to buy 'raised without antibiotics' labelled pork

Our study has shown that the predictive power of the exploratory model was 58%, and that it remained constant when the model was extended with age, gender, education, and SES. Various studies have discovered that sociodemographic factors, particularly age and gender, influence purchase intention. Pelletier et al. (2013) found that age had a significant influence on the purchase of healthy foods and Zhang et al. (2012) similarly identified that younger consumers were more likely to buy traceable food products than other age groups. Gender has also been acknowledged to have an influence on purchase intention, with females having an increased intention to buy traceable pork and organic food (Zhang et al., 2012; Pelletier et al., 2013). In contrast to previous research however, the present study indicates that sociodemographic characteristics have a minimal influence on consumers' willingness to buy 'raised without antibiotics' labelled pork.

In the extended model, behavioural beliefs concerning animal welfare and product quality were the main predictors of willingness to purchase 'raised without antibiotics' labelled pork, followed by pork purchasing habits relating to animal welfare qualities, and perceptions towards personal risks and animal welfare standards, which contributed comparatively less. By contrast, perceptions towards animal antimicrobial use acceptance, 'raised without antibiotics' labelled pork beliefs concerning price, and pork purchasing habits relating to the products extrinsic qualities were found to have a negative influence on consumers' willingness to buy.

Willingness to buy 'raised without antibiotics' labelled pork is mainly driven by behavioural beliefs surrounding product quality and the animal welfare standards associated with production. Conceptually, the influence of beliefs in behaviour formation is consistent with Ajzen's (1991) theory of planned behaviour (TPB), which links beliefs and other core components together to shape an individual's behavioural intentions. Although behavioural beliefs were the only component of this model to emerge from our exploratory study, we would suggest that it merits further investigation through the application of the TPB to identify additional constructs that may drive consumers' purchase intentions towards 'raised without antibiotics' labelled pork.

The present study also shows that perceptions have a significant influence on willingness to buy, with a lack of acceptance towards animal antimicrobial use contributing the most to the model, followed by personal risk of antimicrobial use and concerns for animal welfare. While we are unsure of the reasoning behind consumers' contradictory perceptions towards animal welfare and lacking acceptance of animal antimicrobial use, a possible explanation is outlined in a theory discussed by Grunert (2006), demonstrating the distinction between an individual's role as a consumer and as a citizen. Thus, attitudes towards meat production in their role as a citizen may have limited effect on purchase behaviour as consumers. Individual's may be uncertain or even critical about the consumption of meat from animals given antibiotics but still highly value antimicrobial use as a useful treatment for animal disease. Zingg and Siegrist (2012) also applied this concept to research exploring consumers' perception towards animal vaccination and further detailed that people do not link their acceptance of production attributes (i.e., antibiotic use for disease treatment) with the fact that this meat might enter the food chain in the future.

In relation to consumers' purchase intentions, various other studies have similarly found that such perceptions determine purchasing behaviour (Lobb et al., 2007; Stefani et al., 2008; Kapuge, 2016; Smith et al., 2017). Stefani et al. (2008) found that risk perception was an important determinant of attitude and related to intention to purchase chicken, and similarly, Kapuge (2016) identified that there was a significant impact of health consciousness on purchase intention of organic food among Sri Lankan consumers. In relation to antibiotic research specifically, Smith et al. (2017) uncovered that those who had higher concerns for AMR (i.e., personal risk perception) likely have limited knowledge, which may have prompted them to report negative views towards antimicrobial use in food animal production and, consequently, about intending to purchase antibiotic free meat.

4.5. Implications for the pork industry and animal welfare

As the 'raised without antibiotics' food label is a new addition to the UK market, it is important to understand the consequences that this production has on animal welfare as well as other policy relevant issues, such as food safety and consumer perceptions. As shown in this paper, consumers' purchase intentions are strongly related to their behavioural beliefs of 'raised without antibiotics' labelled pork, and perceptions towards AMR. Our findings also signal that the use of antibiotics in food animal production is negatively related to willingness to buy. Having this understanding of consumer perceptions is essential for the development of communication strategies to address public concern and instil

confidence in the safety of pork (Steede, 2020).

First, the results would seem to indicate that consumers believe antibiotics should not be used in livestock production, suggesting that they are unaware of the negative repercussions antibiotic free production might have on animal welfare. While further analysis is necessary to quantify the detriment associated with animal production free from antibiotics, it is clear that consumers do not possess much knowledge surrounding agricultural antimicrobial use practices and specifically, the role of antibiotics within livestock production. Interventions should therefore focus on addressing this information gap as the elimination of antibiotics can be harmful for animal welfare; however, as consumers lack knowledge this can make risk communication very challenging, as they may not be interested in such information (Zingg & Siegrist, 2012). In addition, it must be taken into account that while consumers generally advocate high animal welfare standards, at the same time, they are less accepting of antimicrobial use in animals. Therefore, agricultural communicators, regulatory bodies, and the UK government have a role to play in information provision to successfully communicate farming practices and the positive role of antibiotics in livestock production. We further suggest that veterinary surgeons are consulted for policy, legislation, and future research as they have an ethical obligation to prevent or treat animal disease and as such, they will not endorse policy whereby necessary antibiotic treatments are withheld.

Furthermore, information should be provided to assure the public about food safety yet provide education of zoonotic diseases and possible routes of transmission, and where appropriate, kitchen hygiene and sufficient cooking to lessen the risk of infection, regardless of how the animal was raised (Smith et al., 2017). In relation to food labelling, particularly regarding the 'raised without antibiotics' label, we suggest that more transparency is provided to consumers. Although the 'raised without antibiotics' label intends to provide additional information to the consumer about how the meat was produced, our study determined that it can be misleading. Organic pork research conducted by Abrams et al. (2010) revealed that US consumers recommended that information is posted in supermarkets, and that it should be clear, concise, and easy to find. This implies that information should be provided directly on food packaging, through the utilisation of a logo or QR code. However, more research is needed in this area to establish the most efficacious route of information provision.

4.6. Study limitations

With respect to the findings discussed above, it is important that the limitations of the study are considered as it may provide scope for future work. Firstly, the current study does not consider different food products. As consumers spend more time purchasing high-involvement products than low-involvement products, the requirement for product information sources may differ depending on the nature of the product (Choi et al., 2010). While antimicrobial use in the pig sector has been highlighted as an area of concern, it would be interesting to explore consumer perceptions towards other meat products (i.e., chicken and beef) to see if their feelings vary towards animal antimicrobial use and the 'raised without antibiotics' label. Specifically, bacon is not typically perceived as a 'health food' thus, it may be possible that concerns surrounding antimicrobial use in pork products may be lower than antimicrobial usage concerns in chicken or other meat products. Secondly, the scale employed in this study to assess participants knowledge of EU regulations was restricted, subsequently, future research may seek to explore both EU and AMR knowledge on a wider scale to gain a more comprehensive understanding of consumer knowledge. As participants self-rated their levels of knowledge and awareness, social desirability bias must also be taken into consideration. Furthermore, the present study was not conducted during a major risk crisis. As many participants were unaware of AMR, they may have never thought about these questions before. However, AMR is a growing problem, with 10 million people estimated to die each year from drug-resistant infections by 2050 (O'Neill, 2016); thus, we predict that public perceptions may change over time as the risk of AMR increases. Subsequently, it is necessary that this research is on-going. Lastly, while this research was exploratory in design, elements of our findings can be linked to the TPB which suggests that it may be of interest for future confirmatory research, with the application of a conceptual framework.

5. Conclusion

Overall, respondents' beliefs on animal welfare and product quality had the greatest influence on their willingness to buy 'raised without antibiotics' labelled pork, followed by their past pork purchasing habits based on animal welfare qualities, and perceptions of antimicrobial use relating to personal concerns and animal welfare standards. Acceptance of animal antimicrobial use, beliefs on price, and consideration of extrinsic qualities when purchasing pork (i.e., price, appearance) had a negative influence on willingness to buy 'raised without antibiotics' labelled pork. Respondents lacked knowledge of AMR and agricultural antimicrobial use practices, and as such they considered the 'raised without antibiotics' food label to be unclear and misleading. Moderate perceptions towards 'raised without antibiotics' labelled pork, lack of knowledge of antimicrobial use and resistance, and the subsequent confusion surrounding this label suggests that it is necessary to explore other labelling options that will educate and provide consumers with the information they desire while also safeguarding animal welfare standards and ethical livestock production. These findings have importance for pork producers, pork labelling, and those involved in the creation of food policy and legislation.

Author contributions

Hollie Bradford wrote the original draft and conducted the formal analyses with the supervision and assistance of Moira Dean. All authors contributed to the conceptualisation, project design and editing of this manuscript and collectively approved the final article.

Ethics statement

This study was approved by the School of Medicine, Dentistry and Biomedical Sciences Faculty Research Ethics Committee, Queen's University Belfast (Faculty REC Reference Number: MHLS 20_23).

Declarations of interest

The authors declare no conflict of interest.

Funding

This research is funded by The Department for the Economy.

Data availability statement

Data was newly acquired for the present study and can be made available after publication.

Acknowledgements

Many thanks to all study authors who contributed to this research.

Appendix A. Supplementary data

Supplementary data to this article can be found online at https://doi.org/10.1016/j.appet.2021.105900.

References

Abrams, K., Meyers, C., & Irani, T. (2010). Naturally confused: Consumers' perceptions of all-natural and organic pork products. *Agriculture and Human Values, 27*(3), 365–374.

Ajzen, I. (1991). The theory of planned behavior. *Organizational Behavior and Human Decision Processes, 50*(2), 179–211.

Bernués, A., Olaizola, A., & Corcoran, K. (2003). Extrinsic attributes of red meat as indicators of quality in europe: An application for market segmentation. *Food Quality and Preference, 14*(4), 265–276.

Bokma, J., Dewulf, J., Deprez, P., & Pardon, B. (2018). Risk factors for antimicrobial use in food-producing animals: Disease prevention and socio-economic factors as the main drivers? *Vlaams Diergeneeskundig Tijdschrift, 87*(4), 188–200.

Bowman, M., Marshall, K., Kuchler, F., & Lynch, L. (2016). Raised without antibiotics: Lessons from voluntary labeling of antibiotic use practices in the broiler industry. *American Journal of Agricultural Economics, 98*(2), 622–642.

Busch, G., Kassas, B., Palma, M., & Risius, A. (2020). Perceptions of antibiotic use in livestock farming in Germany, Italy and the United States. *Livestock Science, 241*, 104251.

Centner, T. (2016). Efforts to slacken antibiotic resistance: Labeling meat products from animals raised without antibiotics in the United States. *The Science of the Total Environment, 563–564*, 1088–1094.

Cervantes, H. (2015). Antibiotic-free poultry production: Is it sustainable? *The Journal of Applied Poultry Research, 24*(1), 91–97.

Choi, Y., Kale, R., & Shin, J. (2010). Religiosity and consumers' use of product information source among Korean consumers: An exploratory research. *International Journal of Consumer Studies, 34*(1), 61–68.

Coyne, L., Latham, S., Dawson, S., Donald, I., Pearson, R., Smith, R., Williams, N., & Pinchbeck, G. (2018). Antimicrobial use practices, attitudes and responsibilities in UK farm animal veterinary surgeons. *Preventive Veterinary Medicine, 161*, 115–126.

Coyne, L., Pinchbeck, G., Williams, N., Smith, R., Dawson, S., Pearson, R., & Latham, S. (2014). Understanding antimicrobial use and prescribing behaviours by pig veterinary surgeons and farmers: A qualitative study. *The Veterinary Record, 175*(23), 593-593.

Denver, S., Sandøe, P., & Christensen, T. (2017). Consumer preferences for pig welfare – can the market accommodate more than one level of welfare pork? *Meat Science, 129*, 140–146.

Driver, A. (2017a). *Antibiotic-free labelling 'very misleading'* [online] Npa-uk.org.uk. Available at: http://www.npa-uk.org.uk/Antibiotic-free_labelling_very_misleading.html. (Accessed 8 January 2021).

Driver, A. (2017b). *New 'raised without antibiotics' label launched* [online] Pig-world.co.uk. Available at: http://www.pig-world.co.uk/news/new-raised-without-antibiotics-label-launched.html. (Accessed 18 January 2021).

Ekakoro, J., Caldwell, M., Strand, E., & Okafor, C. (2019). Drivers, alternatives, knowledge, and perceptions towards antimicrobial use among Tennessee beef cattle producers: A qualitative study. *BMC Veterinary Research, 15*.

Ellison, B., Brooks, K., & Mieno, T. (2017). Which livestock production claims matter most to consumers? *Agriculture and Human Values, 34*(4), 819–831.

Farm Antibiotics. (2021). What does 'antibiotic free' mean? [online] Farmantibiotics.org. Available at: https://www.farmantibiotics.org/ufaqs/antibiotic-free-mean/#:~:text=All%20animal%20farming%20must%20abide,be%20'antibiotic%2Dfree (Accessed 18 January 2021).

Gaucher, M. L., Quessy, S., Letellier, A., Arsenault, J., & Boulianne, M. (2015). Impact of a drug-free program on broiler chicken growth performances, gut health, Clostridium perfringens and Campylobacter jejuni occurrences at the farm level. *Poultry Science, 94*(8), 1791–1801.

Goddard, E., Hartmann, M., & Klink-Lehmann, J. (2017, June). Public acceptance of antibiotic use in livestock production Canada and Germany. In *International European Forum, February 13-17, 2017, 2017* pp. 424–437). Austria: Innsbruck-Igls. No. 1012-2017-709.

Gotschi, E., Vogel, S., Lindenthal, T., & Larcher, M. (2009). The role of knowledge, social norms, and attitudes toward organic products and shopping behavior: Survey results from high school students in Vienna. *The Journal of Environmental Education, 41*(2), 88–100.

Grannis, J., & Thilmany, D. (2001). Marketing natural pork: An empirical analysis of consumers in the mountain region. *Agribusiness, 18*(4), 475–489.

Grunert, K. G. (2006). Future trends and consumer lifestyles with regard to meat consumption. *Meat Science, 74*(1), 149–160.

Grunert, K., Sonntag, W., Glanz-Chanos, V., & Forum, S. (2018). Consumer interest in environmental impact, safety, health and animal welfare aspects of modern pig production: Results of a cross-national choice experiment. *Meat Science, 137*, 123–129.

Gulab, S. (2018). *Understanding consumer attitudes towards antimicrobial risk reducing practices. [pdf] lincoln, Nebraska: Dissertations and theses in agricultural economics*. Available at: http://digitalcommons.unl.edu/agecondiss/51. (Accessed 6 January 2021).

Howell, T., Rohlf, V., Coleman, G., & Rault, J. (2016). Online chats to assess stakeholder perceptions of meat chicken intensification and welfare. *Animals, 6*(11), 67.

Hughner, R., McDonagh, P., Prothero, A., Shultz, C., & Stanton, J. (2007). Who are organic food consumers? A compilation and review of why people purchase organic food. *Journal of Consumer Behaviour, 6*(2–3), 94–110.

Inglis, V., Ball, K., & Crawford, D. (2005). Why do women of low socioeconomic status have poorer dietary behaviours than women of higher socioeconomic status? A qualitative exploration. *Appetite, 45*(3), 334–343.

Jaja, O. (2017). *A survey of public knowledge and attitude related to antibiotic use and antibiotic resistance in Southwest Alberta (Doctoral dissertation, Lethbridge, Alta*. University of Lethbridge, Dept. of Health Sciences.

Kapuge, K. D. L. R. (2016). Determinants of organic food buying behavior: Special reference to organic food purchase intention of Sri Lankan customers. *Procedia food science, 6*, 303–308.

Karavolias, J., Salois, M., Baker, K., & Watkins, K. (2018). Raised without antibiotics: Impact on animal welfare and implications for food policy. *Translational Animal Science, 2*(4), 337–348.

Kline, P. (1999). *The handbook of psychological testing* (2nd ed.). London: Routledge.

Lobb, A. E., Mazzocchi, M., & Traill, W. B. (2007). Modelling risk perception and trust in food safety information within the theory of planned behaviour. *Food Quality and Preference, 18*(2), 384–395.

Lusk, J., Norwood, F., & Pruitt, J. (2006). Consumer demand for a ban on antibiotic drug use in pork production. *American Journal of Agricultural Economics, 88*(4), 1015–1033.

McCarthy, M., O'Reilly, S., Cotter, L., & de Boer, M. (2004). Factors influencing consumption of pork and poultry in the Irish market. *Appetite, 43*(1), 19–28.

O'Neill, J. (2016). Tackling drug-resistant infections globally: Final report and recommendations. *Review on Antimicrobial Resistance*, 1–84.

Olynk, N. J., Tonsor, G. T., & Wolf, C. A. (2010). Consumer willingness to pay for livestock credence attribute claim verification. *Journal of Agricultural and Resource Economics, 35*, 261–280.

Pelletier, J. E., Laska, M. N., Neumark-Sztainer, D., & Story, M. (2013). Positive attitudes toward organic, local, and sustainable foods are associated with higher dietary quality among young adults. *Journal of the Academy of Nutrition and Dietetics, 113*(1), 127–132.

Rahnama, H., Fadaei, M., & Baghersalimi, S. (2017). Healthy food choice: Survey results from Iranian consumers toward antibiotic-free chicken. *Journal of Sensory Studies, 32*(1), e12248.

RUMA. (2016). 'Antibiotic-free' labelling [online] Responsible Use of Medicines in Agriculture Alliance. Available at: https://www.ruma.org.uk/ruma-confirms-position-on-antibiotic-free-labelling/. (Accessed 15 January 2021).

Sadiq, M., Syed-Hussain, S., Ramanoon, S., Saharee, A., Ahmad, N., Mohd Zin, N., Khalid, S., Naseeha, D., Syahirah, A., & Mansor, R. (2018). Knowledge, attitude and perception regarding antimicrobial resistance and usage among ruminant farmers in Selangor, Malaysia. *Preventive Veterinary Medicine, 156*, 76–83.

Sanders, D. R., Moon, W., & Kuethe, T. H. (2007). Consumer willingness-to-pay for fresh pork attributes. *Journal of Agribusiness, 25*, 163–179, 345–2016-15148.

Schroeder, J., Chassy, B., Tribe, D., Brookes, G., & Kershen, D. (2014). Organic marketing report [pdf] Available at: https://hoards.com/sites/default/files/Academics-Review_Organic-Marketing-Report.pdf. (Accessed 8 January 2021).

Singer, R., Porter, L., Thomson, D., Gage, M., Beaudoin, A., & Wishnie, J. (2019). Raising animals without antibiotics: U.S. Producer and veterinarian experiences and opinions. *Frontiers in Veterinary Science, 6*.

Smith, R. A., Zhu, X., Shartle, K., Glick, L., & M'ikanatha, N. M. (2017). Understanding the public's intentions to purchase and to persuade others to purchase antibiotic-free meat. *Health Communication, 32*(8), 945–953.

Spence, M., Stancu, V., Elliott, C. T., & Dean, M. (2018). Exploring consumer purchase intentions towards traceable minced beef and beef steak using the theory of planned behavior. *Food Control, 91*, 138–147.

Steede, G., Meyers, C., Li, N., Irlbeck, E., & Gearhart, S. (2020). The influence of framing effects on public opinion of antibiotic use in livestock. *Journal of Applied Communications, 104*(2).

Stefani, G., Cavicchi, A., Romano, D., & Lobb, A. E. (2008). Determinants of intention to purchase chicken in Italy: The role of consumer risk perception and trust in different information sources. *Agribusiness: International Journal, 24*(4), 523–537.

Tang, K., Caffrey, N., Nóbrega, D., Cork, S., Ronksley, P., Barkema, H., Polachek, A., Ganshorn, H., Sharma, N., Kellner, J., & Ghali, W. (2017). Restricting the use of antibiotics in food-producing animals and its associations with antibiotic resistance in food-producing animals and human beings: A systematic review and meta-analysis. *The Lancet Planetary Health, 1*(8), e316–e327.

Tunsagool, P., Mhuantong, W., Tangphatsornruang, S., Am-In, N., Chuanchuen, R., Luangtongkum, T., & Suriyaphol, G. (2021). Metagenomics of antimicrobial and heavy metal resistance in the cecal microbiome of fattening pigs raised without antibiotics. *Applied and Environmental Microbiology, 87*(8).

Umberger, W., Boxall, P., & Lacy, R. (2009). Role of credence and health information in determining US consumers' willingness-to-pay for grass-finished beef. *The Australian Journal of Agricultural and Resource Economics, 53*(4), 603–623.

Ursachi, G., Horodnic, I., & Zait, A. (2015). How reliable are measurement scales? External factors with indirect influence on reliability estimators. *Procedia Economics and Finance, 20*, 679–686.

Verain, M., Bartels, J., Dagevos, H., Sijtsema, S., Onwezen, M., & Antonides, G. (2012). Segments of sustainable food consumers: A literature review. *International Journal of Consumer Studies, 36*(2), 123–132.

Verbeke, W., & Liu, R. (2014). The impacts of information about the risks and benefits of pork consumption on Chinese consumers' perceptions towards, and intention to eat, pork. *Meat Science, 98*(4), 766–772.

Wardle, J., Haase, A. M., Steptoe, A., Nillapun, M., Jonwutiwes, K., & Bellisie, F. (2004). Gender differences in food choice: The contribution of health beliefs and dieting. *Annals of behavioral medicine, 27*(2), 107–116.

Wheatley, W. P. (2001). *Consumer preferences, premiums, and the market for natural and organic pork: Locating a niche for small-scale producers*. North Mankato, MN: Minnesota Pork Producers Association.

Yang, W., & Renwick, A. (2019). Consumer willingness to pay price premiums for credence attributes of livestock products – a meta-analysis. *Journal of Agricultural Economics, 70*(3), 618–639.

Yiridoe, E. K., Bonti-Ankomah, S., & Martin, R. C. (2005). Comparison of consumer perceptions and preference toward organic versus conventionally produced foods: A review and update of the literature. *Renewable Agriculture and Food Systems*, 193–205.

Zhang, C., Bai, J., & Wahl, T. (2012). Consumers' willingness to pay for traceable pork, milk, and cooking oil in Nanjing, China. *Food Control, 27*(1), 21–28.

Zingg, A., & Siegrist, M. (2012). People's willingness to eat meat from animals vaccinated against epidemics. *Food Policy, 37*(3), 226–231.
